# Towards Substrate–Reagent Interaction of Lochmann–Schlosser Bases in THF: Bridging THF Hides Potential Reaction Site of a Chiral Superbase

**DOI:** 10.1002/chem.202202660

**Published:** 2022-10-17

**Authors:** Lukas Brieger, Tobias Schrimpf, Rebecca Scheel, Christian Unkelbach, Carsten Strohmann

**Affiliations:** ^1^ Inorganic Chemistry TU Dortmund University Otto-Hahn-Str. 6/6a 44227 Dortmund Germany

**Keywords:** alkali metals, chirality, lithium, potassium, X-ray diffraction

## Abstract

The metalation of *N*,*N*‐dimethylaminomethylferrocene in THF by the superbasic mixture of ^*n*^BuLi/KO^*t*^Bu proceeds readily at low temperatures to afford a bimetallic Li_2_K_2_ aggregate containing ferrocenyl anions and *tert*‐butoxide. Starting from an enantiomerically enriched *ortho*‐lithiated aminomethylferrocene, an enantiomerically pure superbase can be prepared. The molecular compound exhibits superbasic behavior deprotonating *N*,*N*‐dimethylbenzylamine in the *α*‐position and is also capable of deprotonating toluene. Quantum chemical calculations provide insight into the role of the bridging THF molecule to the possible substrate–reagent interaction. In addition, a benzylpotassium alkoxide adduct gives a closer look into the corresponding reaction site of the Lochmann–Schlosser base that is reported herein.

## Graphical abstract

The isolation of a new THF‐solvated, ferrocene‐based Lochmann–Schlosser base with all four components lithium, potassium, carbanion, and alkoxide led to the observation of new structural motifs with respect to these superbasic reagents. The “kick out” of a THF leads to the detachment, providing the typical Li/K reaction site, whereas the “smart pass” leads to the favorable THF‐displacement and the K/K reaction site provided for substrates.

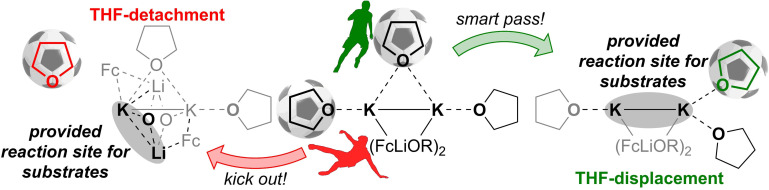

## Introduction

**Figure 1:**
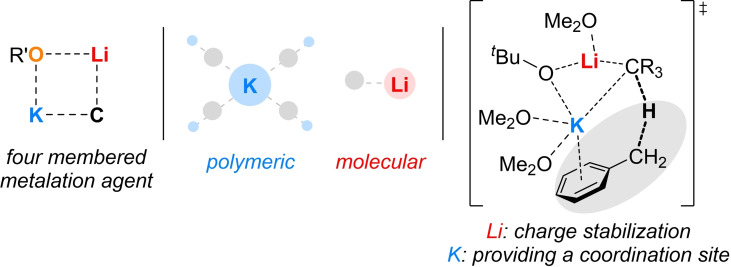
Initial suggestion of the Lochmann–Schlosser base's key metalation agent and the different roles of its alkali metals; R=H, Alkyl.

Deprotonations with the Lochmann–Schlosser base^[1]^ have become indispensable and an enormously important tool in organometallic chemistry since the independent discoveries of their namesakes and in the context of alkali metal mediation chemistry.^[2]^ For example, metals can be introduced to influence and exploit the nature of the anion^[3]^ or to control the positions of the metalation to synthesize pharmaceuticals.^[4]^ The mechanism of deprotonation is still the subject of current research. Initial studies by Schlosser assumed a four‐membered metalation agent^[1b]^ which interacts with the substrate (Figure 1). A first model for a superbase was provided by Harder and Streitwieser with the isolation of a bimetallic organosodium‐lithium alkoxide compound.^[5]^ 20 years later, with the metalation of benzene, a first reactive intermediate of a Schlosser base with all its components was isolated and characterized.^[6]^ It was shown that the aggregate itself deprotonates toluene and thus acts like a superbase.

**Scheme 1:**
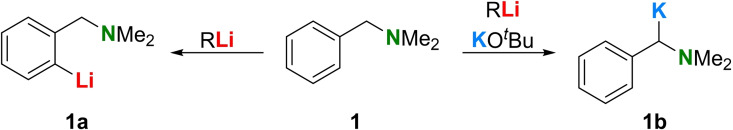
Deprotonation of DBA with organolithium compounds (left) and with a Lochmann–Schlosser base (right); R=Alkyl.

Key for the deprotonation to proceed was the detachment of a THF molecule coordinated to a potassium center in exchange for the coordination of the substrate, which provided an initial suggestion for the mechanism in THF. It also turned out that the two metals have different but specific roles in the aggregate itself, but also in subsequent deprotonation reactions. Lithium is crucial for the charge stabilization through short, strong interactions to the anions present and the molecular structure, whereas potassium forms weak interactions to mainly solvents and *π*‐systems, which further supports the proposed reaction mechanism. With the alkane‐soluble superbase of Klett et al., formed by neopentyllithium and potassium‐*tert*‐butoxide further insights into the deprotonation with the bimetallic base mixtures were gained.^[7]^ In addition to the structure of the bimetallic mixtures, their application is also of enormous importance, as the superbasic mixture enables reactions which cannot be achieved by their individual components. For example, the deprotonation of *N*,*N*‐dimethylbenzylamine (DBA) with organolithium reagents always occurs in the *ortho*‐position due to the directing metalation group (DMG),^[8]^ but metalation with the bimetallic Lochmann–Schlosser base leads to the deprotonation of the benzylic carbon center, which enables the possibility of functionalization in the benzylic position (Scheme 1).^[9]^ In this context, stereogenic centers could also be established, because stereoselective deprotonation and subsequent functionalization could be used to specifically address one of the two protons and replace it with a functional group. This would require a suitable deprotonation reagent, since so far mainly alkyl lithium reagents in combination with chiral amine ligands have been used in synthetic chemistry,^[10]^ which in turn would lead to the formation of the undesired *ortho*‐product, regarding the deprotonation of *N*,*N*‐dimethylbenzylamine.

## Results and Discussion

**Scheme 2:**
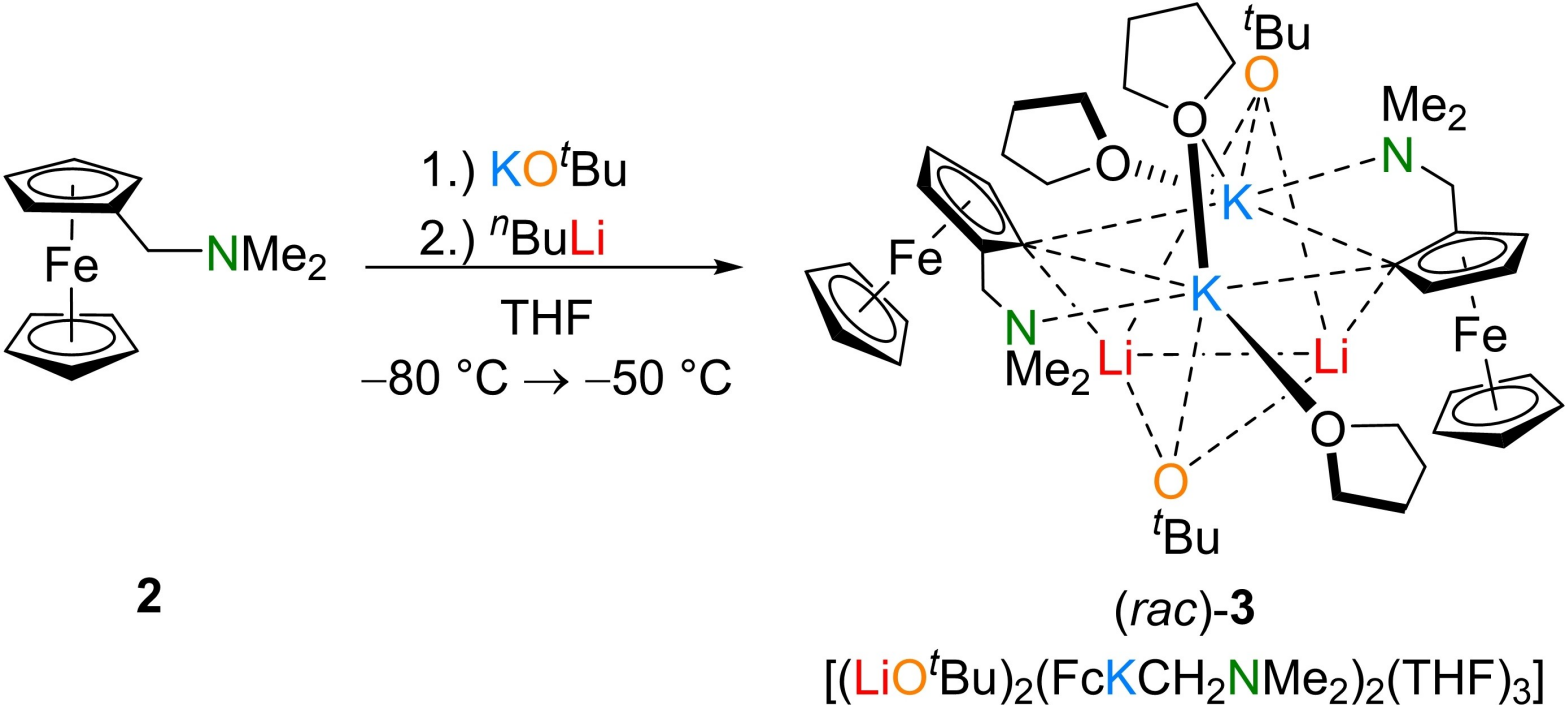
Racemic approach for the synthesis of (*rac*)‐**3**.

Herein, we report on the formation of the first crystalline, chiral superbase and its contribution to mechanistical investigations of reactions of superbases with substrates in THF. Moreover, the capability to deprotonate toluene and *N*,*N*‐dimethylbenzylamine is demonstrated. Treatment of ^*n*^BuLi/KO^*t*^Bu in THF at −80 °C with *N*,*N*‐dimethylaminomethylferrocene (**2**) afforded an orange‐red solution immediately, which was subjected to crystallization studies. Storage of the metalated compound in solution at −80 °C resulted in the formation of yellow blocks, which turned out to be aggregate **3** (Scheme 2). The intermediate crystallizes from THF in the monoclinic crystal system, space group *C*2. The asymmetric unit contains half of the molecule as well as a co‐crystallized THF‐molecule.

**Figure 2:**
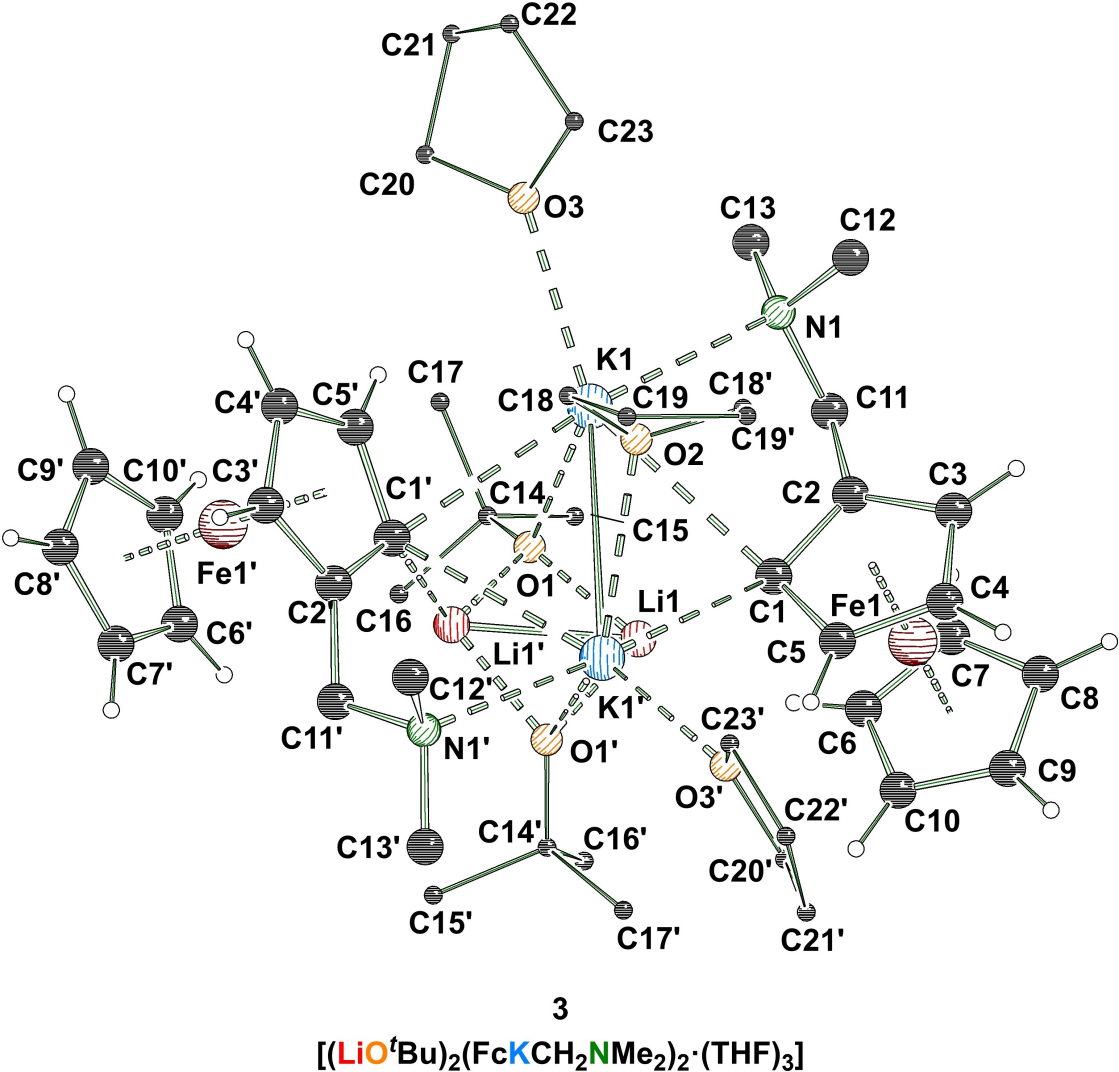
Molecular structure of [(LiO^*t*^Bu)_2_(FcKCH_2_NMe_2_)_2_(THF)_3_] (**3**; some hydrogen atoms and disorders are omitted for clarity). Symmetry‐equivalent positions are included to visualize the coordination environment of the metal centers (#1’: 1−x, y, 1−z). For further information see Supporting Information.

Surprisingly, the composition of **3** was [(LiO^*t*^Bu)_2_(FcKCH_2_NMe_2_)_2_(THF)_3_] and thus containing all components of a superbase in a 1 : 1 ratio. As in the examples of superbasic aggregates known from literature, also in **3** a four‐membered ring, containing two lithium centers and two *tert*‐butoxide anions, is found as the central unit. Superimposed on this motif is a second four‐membered (KC)_2_‐ring formed by two potassium centers and two ferrocenyl anions. The coordination sphere of the potassium centers is saturated by the nitrogen atoms of the aminomethyl handles and by the coordination of the oxygen atoms of the *tert*‐butoxides and two THF molecules each, with one THF molecule bridging the two metal cations. The two four‐membered rings are twisted 90° to each other with the metalated carbon centers pointing towards the lithium centers, thus ensuring the greatest possible interaction between anions and cations with typical C−Li contacts of 2.158(12) Å and 2.144(11) Å.^[11]^ The potassium‐containing ring features typical C−K bonds, between 3.114(6) Å and 3.204(6) Å, as well (Figure 2).^[6]^

In this context, the next step was to evaluate the superbasic properties of this compound by deprotonating toluene. Reaction of (*rac*)‐**3** with toluene leads to the formation of a strong red solution, which becomes colorless after reaction with chloro(trimethyl)silane. Analysis of the crude product via GC/EI‐MS and NMR spectroscopy shows the formation of the functionalized toluene, whereas the metalated ferrocene from aggregate **3** was not functionalized. These results lead to the assumption that **3** quantitatively deprotonates toluene. Due to the positive results in the deprotonation reaction of toluene, another substrate, *N*,*N*‐dimethylbenzylamine **1** (DBA), was tested. In a first reaction (*rac*)‐**3** was used to check the deprotonation of the amine **1**, which turned out to deprotonate regioselectively the benzyl position in a quantitative yield.

### Can we translate the new structural motifs into a reaction mechanism?

Further, compound **3** exhibits a new structural peculiarity not previously seen in comparable superbasic aggregates even though *tert*‐butoxide ligands play a major role in mixed alkoxide aggregates.^[12]^ The peculiarity of this compound involves a bridging THF molecule, which is a generally known structural motif in THF‐soluble stable organopotassium products,^[13]^ but a previously unknown structural motif in reagent structures of superbasic systems. In this context it represents a new structural element to explain to what extent THF‐solvated Lochmann–Schlosser bases might react. From previous studies, we have already learned that the pre‐coordination of the substrate plays a crucial role, which in this example was initiated by a prior detachment of the THF molecule. In this process, as mentioned before, the metals lithium and potassium play different but specific roles that stabilize corresponding transition states, but are also important in explaining the mechanism.

**Figure 3:**
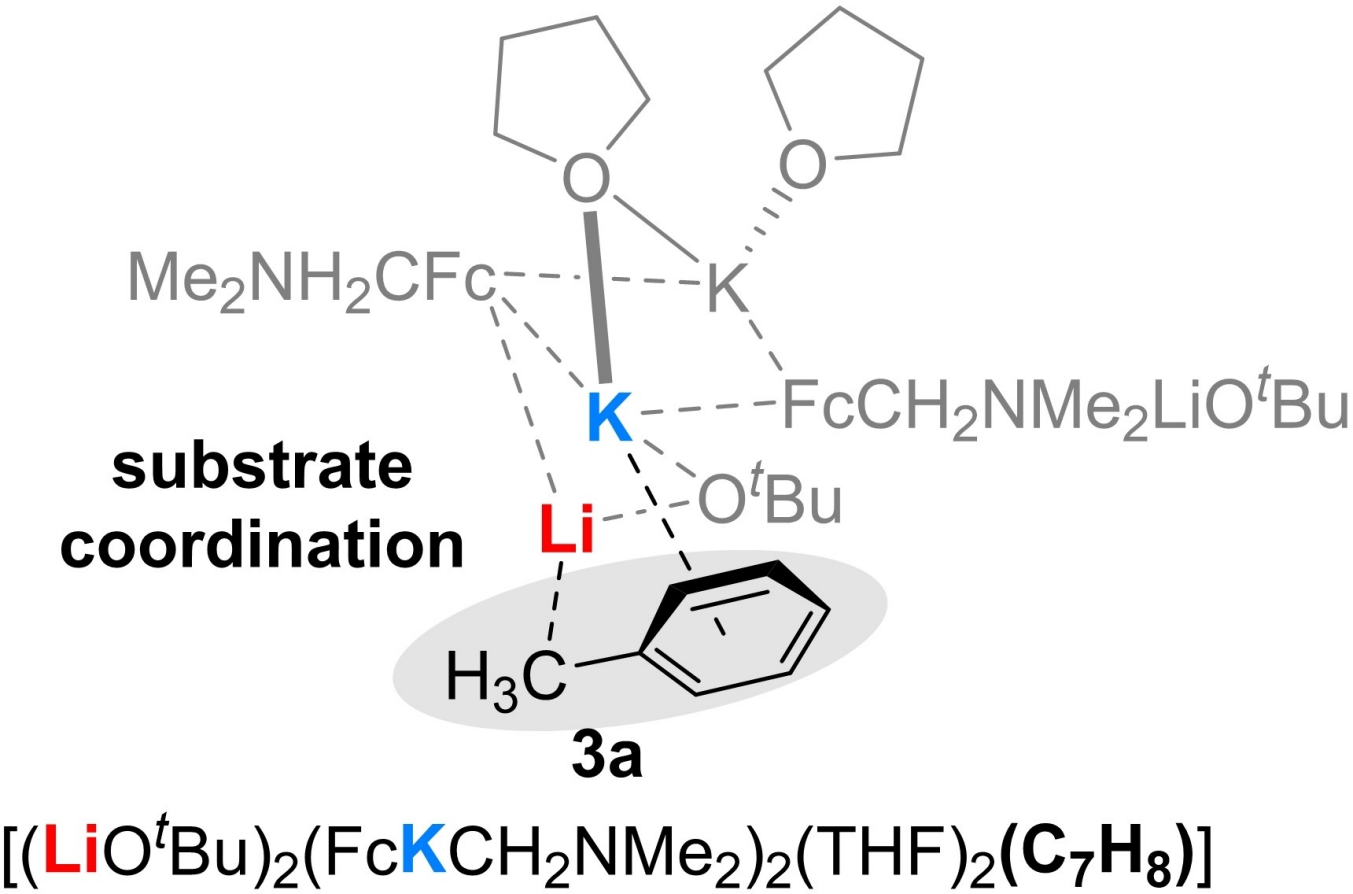
Possible pre‐coordination of toluene after a THF side‐detachment in compound **3**.

**Figure 4:**
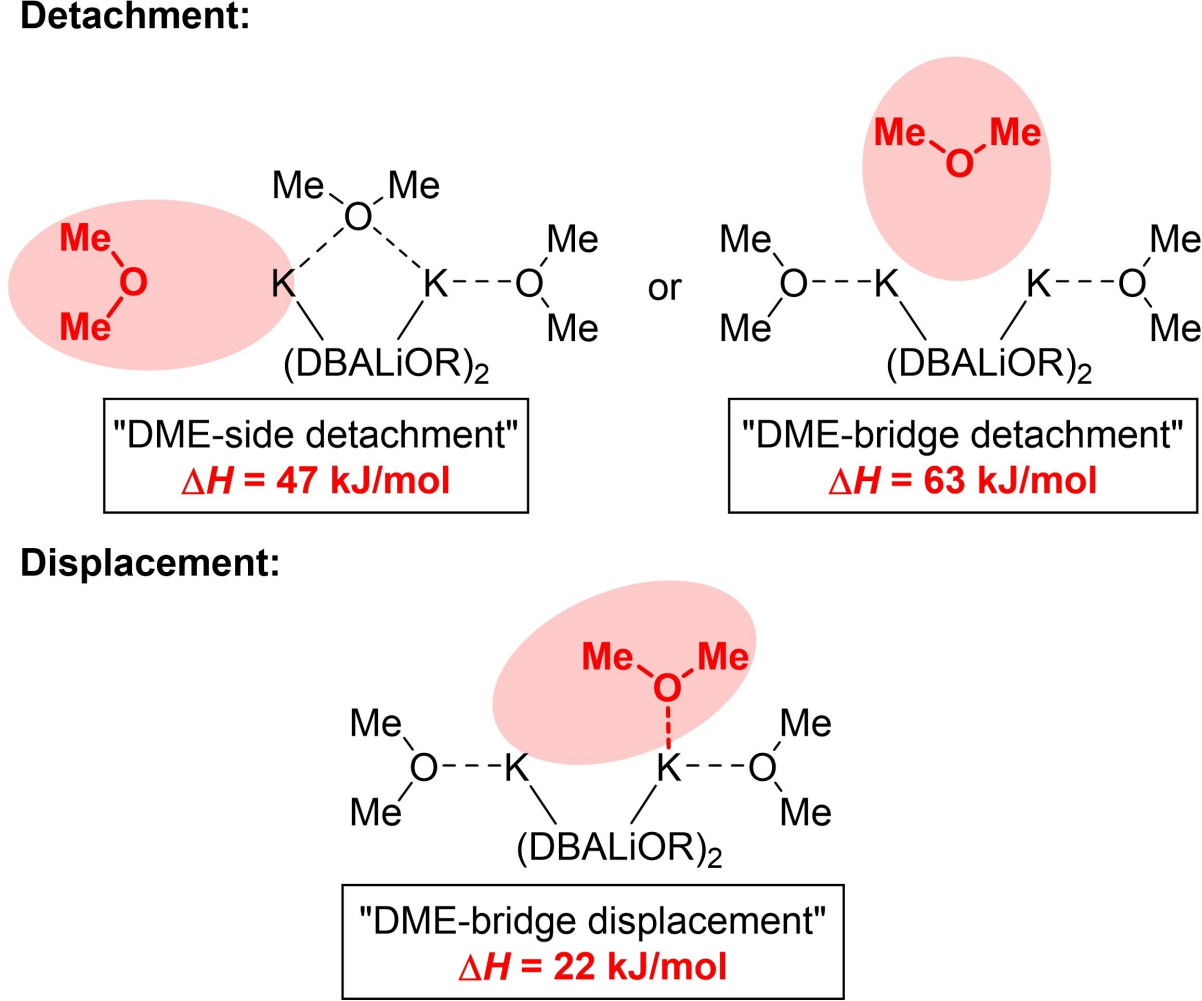
Model of the different mechanisms how the THF molecules (simplified using DME instead of THF and DBA instead of FcCH_2_NMe_2)_ in **3** could detach from the potassium centers and the corresponding calculated energy values. For more information see QM7–QM13 in the Supporting Information.

First, we wanted to understand to what extent different THF detachment mechanisms in compound **3** could play a role in initiating the substrate coordination (Figure 3). Based on the molecular structure in the solid state, we were able to first energetically optimize a simplified ground state with C2‐symmetry on the M062X/def2svp level of theory and then calculate a detachment of a dimethylether (DME) molecule from the side and from the bridge. It was shown that the detachment of the “side” DME molecule from the corresponding potassium center is energetically more favorable (47 kJ/mol) than the detachment of the bridging DME molecule from both potassium centers with 63 kJ/mol (Figure 4).^[13]^

**Figure 5:**
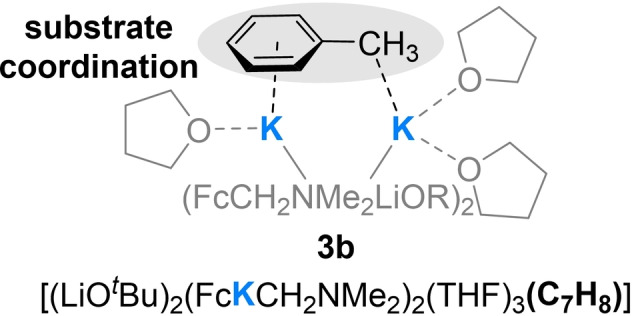
Possible pre‐coordination of toluene after a THF displacement in compound **3**.

In addition to the already known mechanism of a possible detachment of a coordinating THF molecule, compound **3** now offers a new and alternative mechanism to explain the coordination of a substrate to such superbasic intermediates. The bridging THF molecule could shift toward a potassium center, providing a coordination site for substrates. Unlike the previous reaction site, this one does not consist of two different alkali metals lithium and potassium, but of two potassium centers interacting with the substrate (Figure 5).

**Scheme 3:**
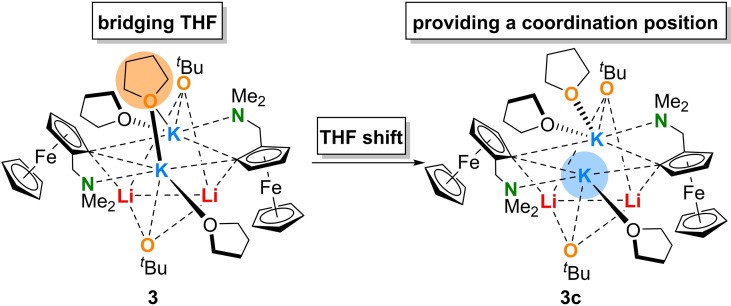
Presentation of the energetically preferred THF displacement within **3**.

Therefore, we aimed to contribute to a better understanding of the mechanism by further quantum chemical calculations on the M062X/def2svp level of theory. As in the case of the detachment, the displacement of the bridging DME should be compared with the initial structure. The displacement was calculated using a relaxed potential energy surface (PES) scan with geometry optimization at each point. Accordingly, the displacement of the bridging DME molecule was found to cost 22 kJ/mol. However, compared to the DME detachment, the displacement is energetically more favorable by 25 kJ/mol and 41 kJ/mol, respectively, and thus should proceed preferentially. Nevertheless, these processes can also occur simultaneously, and these calculations only provide a first idea of which mechanism should proceed preferentially. The lateral detachment of the DME was also observed via a relaxed PES scan. Depending on the distance of the detaching DME molecule from the potassium center, the displacement of the DME molecule should be energetically more preferential with increasing distance (Scheme 3).

**Figure 6:**
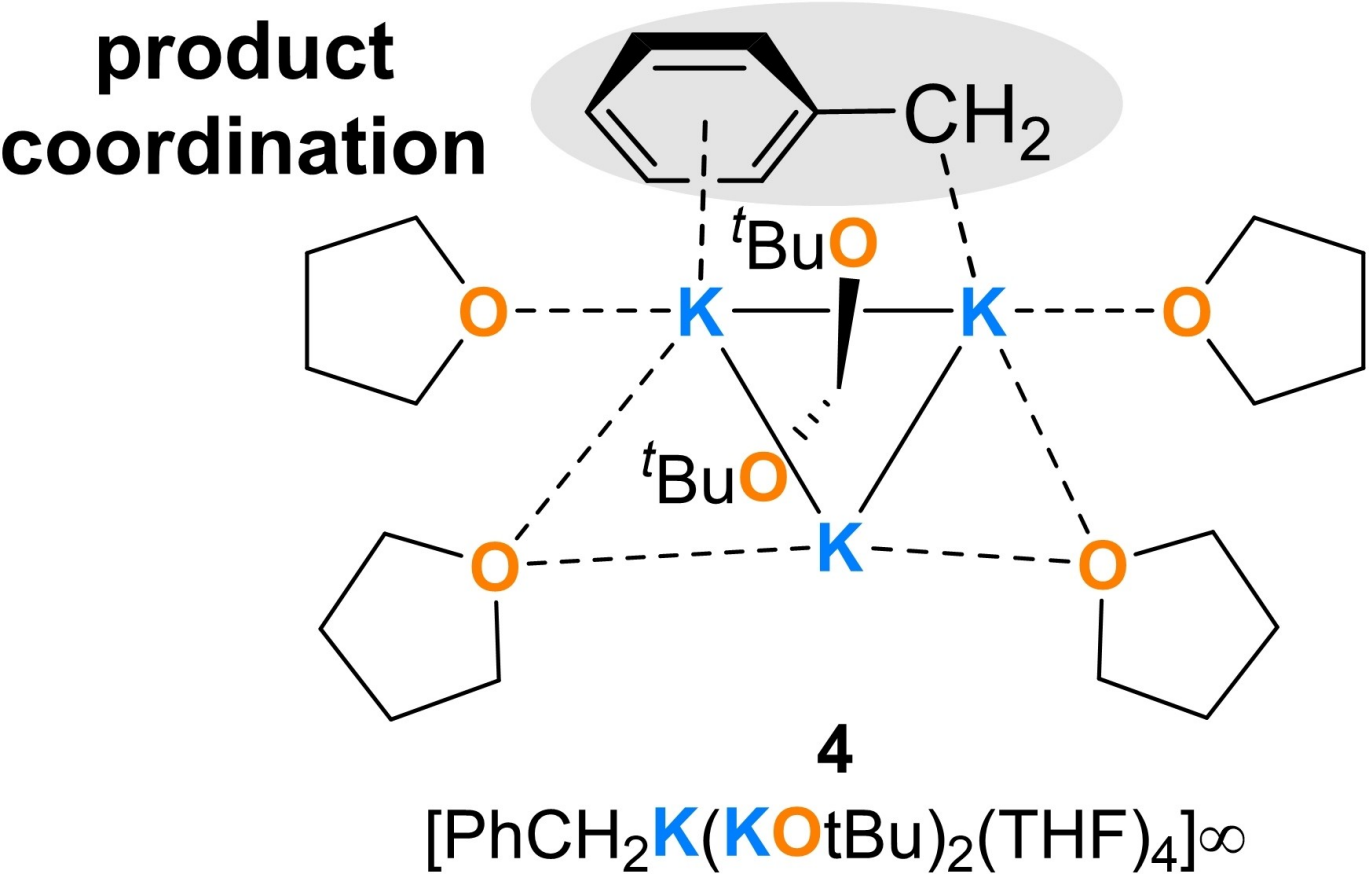
Lewis formula of compound **4** representing the central structural motif within the crystal structure, which contains a benzyl anion that interacts with two potassium centers. The lines between the potassium cations are to clarify the structural motif.

Currently, we are looking for suitable simple models to describe such compounds and suitable transition states, since, as already in the presented quantum chemical models, some simplifications have to be made and a present symmetry simplifies the systems. However, this is beyond the scope of the project presented here, which merely attempts to establish a general concept of a mechanism. In the course of further studies by our group on the deprotonation of toluene with Lochmann–Schlosser bases, a part of which has been published recently, a compound could be obtained with a ratio of ^*n*^BuLi to KO^*t*^Bu of 1 : 3, in which a benzyl anion interacts with two potassium centers and thus forms a similar structural motif as shown in Figure 6.^[15]^ Nevertheless, by isolating and characterizing compound **4**, we were able to show that compounds from a previous pre‐coordination to a K–K system and subsequent deprotonation exist.[^16^, ^17^]

### To what extent is it possible to prepare enantiomerically pure potassium compounds?

**Scheme 4:**
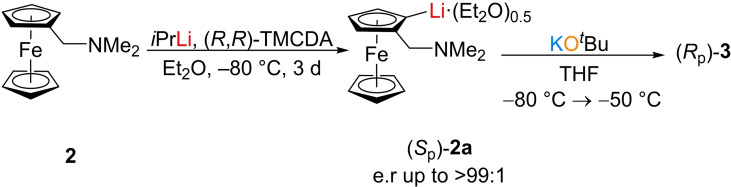
Stereoselective approach for the synthesis of (*R*_p_)‐**3**.

In addition to the superbasic properties of compound **4**, it also potentially represents one of the first ever enantiomeric pure organopotassium compounds structurally characterized. First of all, compound **3** crystallizes in a chiral space group (*C*2, sohnke space group) and further both ferrocenes in aggregate **3** are deprotonated in the same *ortho*‐position, which should result in a *R*_p_‐configurated product in this crystal. With respect to the entire product, a racemic compound is present, since the starting material was racemic. Starting from enantiomerically pure lithiated ferrocene,^[11]^ potassium tert‐butoxide was added to form the aggregate with only one absolute configuration (Scheme 4).

This approach indeed led to the desired enantiomerically pure aggregate (*R*_p_)‐**3** and thus to the first structurally elucidated enantiomerically pure Lochmann–Schlosser base. As the reaction of (*R*_p_)‐**3** with chloro(trimethyl)silane did not show racemization, the isolation of the superbasic reagent now provides potential access to additional enantiomerically enriched organo‐potassium compounds via kinetically controlled deprotonation. The prerequisite for this is that the resulting potassium compound must have a stable configuration so that the chiral information is retained in a substitution reaction. A suitable substrate is *N*,*N*‐dimethylbenzylamine (DBA), which carries a stereogenic carbon center in the benzylic position. In this context, we were able to get insights into the metalation of *N*,*N*‐dimethylbenzylamine with a Lochmann–Schlosser base. Reaction of the amine with a bimetallic mixture of *n*‐butyllithium and potassium *tert*‐butoxide in THF resulted in the formation of a dark red solution from which a crop of block‐shaped red crystals formed after storage at −80 °C. After the single crystal X‐ray structural analysis was carried out, the crystals were found to be compound [PhCHNKMe_2_⋅(THF)_2_] (**5**). The potassiated *N*,*N*‐dimethylbenzylamine forms a zig‐zag like 1D‐coordination polymer in the solid state. The potassium center exhibits η^3^‐ and η^6^‐contacts to a total of two benzyl anions, while three additional σ^1^‐coordinations of two oxygen centers through attached THF molecules and of a nitrogen center of the aminomethyl handle saturate the coordination sphere (Figure 2).

**Figure 7:**
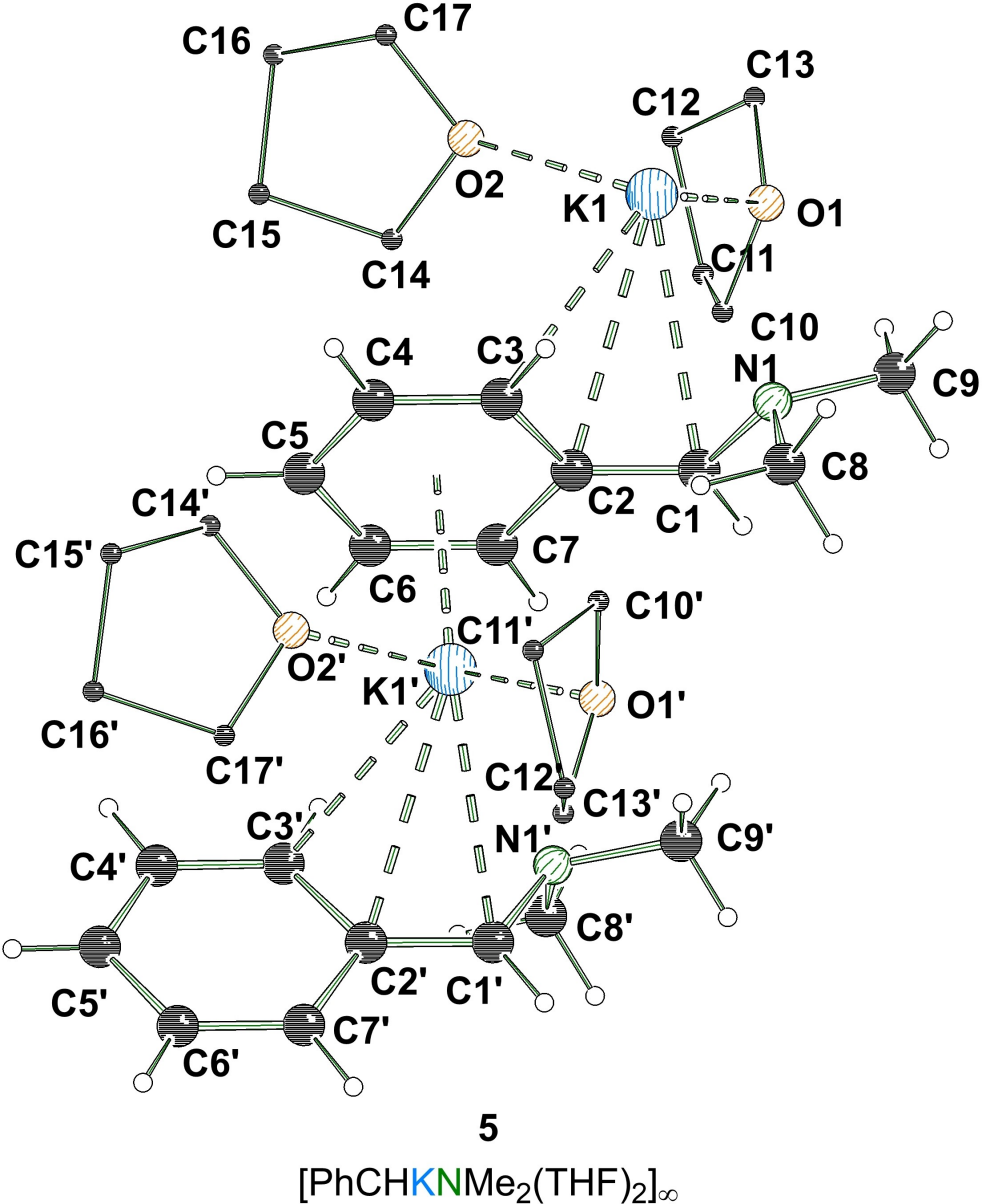
Polymeric structure of [(PhCHKNMe_2_)(THF)_2_]_∞_ (**5**; some hydrogen atoms and disorders are omitted for clarity). Symmetry‐equivalent positions are included to visualize the coordination environment of the potassium centers (#1’: +x, 3/2−y, 1/2+z). For further information see Supporting Information.

It is particularly striking that the metalated carbon center is strongly planarized [∑_(C1)_=359.49(3)°], which favors a delocalization of the negative charge. The benzylanion formed demonstrates typical geometric changes that support the hypothesis for a strong delocalization of the negative charge. With a bond length of 1.380(1) Å, the C_*α*_−C_*ipso*_ bond is strongly shortened as well as the C_*ortho*_−C_*meta*_ bonds. In contrast, the C_*ipso*_−C_*ortho*_‐ and C_*meta*_−C_*para*_‐bonds are elongated. The planar geometry of the metalated carbon center further helps to explain the labile configuration, as the electrophilic attack on the benzylic carbon center can occur either from above or below.

**Scheme 5:**
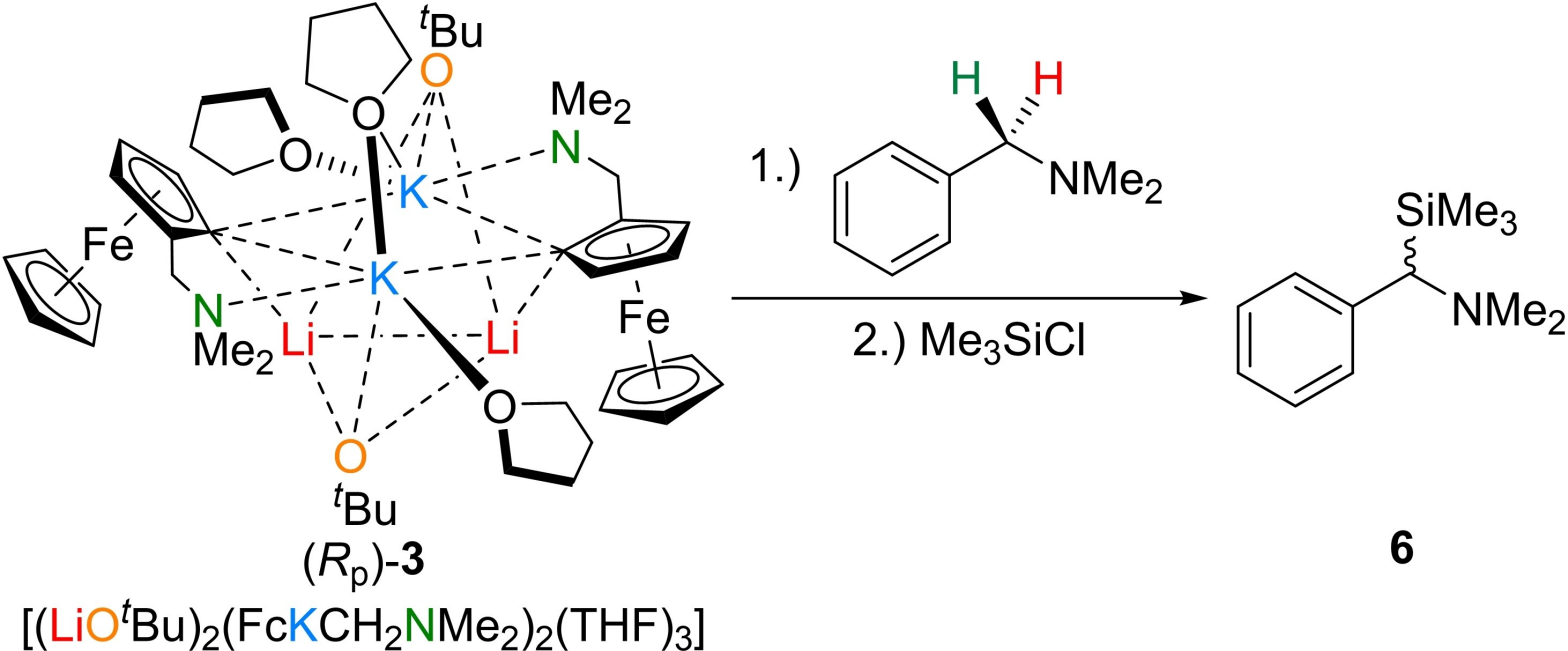
Deprotonation of **1** with (*R*_p_)‐**3**.

Therefore, the deprotonation reaction was carried out again with the enantiomerically pure reagent (*R*_p_)‐**3** in view of whether stereoselective deprotonation reactions are feasible with this compound. To ensure a stable configuration, a very low reaction temperature should be considered. In general, this is not a problem due to the high reactivity of Lochmann–Schlosser bases, but the superbase used here showed no conversion at −80 °C, so that the deprotonation was carried out in THF at −50 °C for 2 h and subsequently trapped with chloro(trimethyl)silane. Again, no silylated ferrocene but only silylated amine was observed by GC/EI‐MS and NMR spectroscopy. However, the far more important question regarding stereoselective deprotonation could only be answered by a further NMR study in combination with (*R*)‐mandelic acid, which resulted in an enantiomeric ratio of 1 : 1 (*e*. *r*.=1 : 1). Hence, no asymmetric induction by deprotonation was observed using the enantiomeric pure Lochmann–Schlosser base at −50 °C for the metalation of DBA (Scheme 5).

Ultimately, these results show that potassium compounds can be prepared using the ferrocene‐based Lochmann–Schlosser base, but the configuration is not stable in this case. Nevertheless, other systems may prove to be more targetable.

## Conclusion

We have demonstrated the directed and selective synthesis of a ferrocene‐based Lochmann–Schlosser base capable of deprotonating substrates to prove the superbasic behavior. The structural feature of a bridging THF molecule serves as an explanatory approach for the interaction between substrate and reagent of formed Lochmann–Schlosser base systems in THF, providing a previously undiscussed reaction site of two potassium centers. Furthermore, we were able to isolate an exemplary compound for this unregarded reaction site. Meanwhile, the planar chirality of the aminomethylferrocenes used allows the isolation of an enantiomerically pure Lochmann–Schlosser base, even though the enantioselective deprotonation of *N*,*N*‐dimethylbenzylamine (**1**) could not be successfully carried out with them to date. In this context, we were able to gain further insight into the deprotonation of DBA by isolating a metalated species, which explains the non‐stereospecific reaction.

## Experimental Section

**General Remarks**: All reactions with oxygen‐ and moisture‐sensitive compounds were performed under an atmosphere of argon in dried solvents, which were distilled prior to use. All other solvents and commercially available reagents, including the NMR solvents, were used without further purification. The NMR spectra were measured on a *Bruker Avance DRX‐400* and on a *Bruker Avance DRX‐500* NMR spectrometer. All NMR spectra were recorded at room temperature (ca. 22 °C). Chemical shifts (*δ* in ppm) are referred to tetramethylsilane (TMS), with the deuterium signal of the solvent serving as internal lock and the residual solvent signal as additional Ref. [^1^H NMR: *δ*(C_6_D_5_H)=7.16, *δ*(C_4_D_7_ *H*O)=1.73; 3.58, *δ*(C*H*Cl_3_)=7.26; ^13^C NMR: *δ*(*C*_6_D_6_)=128.4, *δ*(*C*_4_D_8_O)=25.3; 67.2, *δ*(*C*DCl_3_)=77.0]. The ^29^Si NMR experiments are referred to TMS as external standard and measured via the INEPT pulse sequence. For the assignment of the multiplicities the following abbreviations were used: s=singlet, d=doublet, t=triplet, m=multiplet, br=broad signal. Aromatic carbon and hydrogen atoms were assigned as follows: i=ipso, o=ortho, m=meta, p=para. GC/EI‐MS analyses were obtained using an Agilent 7890B GC system (column: Agilent HP‐5MS, 30 m, 0.25 mm, 0.25 μm) with an Agilent 5977 A Mass Selective Detector. Suitable crystals of compound (*rac*)‐**3**, (*R*_p_)‐**3**, **4** and **5** were covered with an inert oil (perfluoroalkylether) at −80 °C using the *X‐TEMP 2* ^[1]^ device in combination with a *SMZ1279* stereomicroscope from *Nikon Metrology GmbH* and mounted on a *MicroMount* from *MiTeGen*. Crystal structure determination was accomplished on a *Bruker D8 Venture* four‐circle diffractometer using a *PHOTON II CPAD* detector by *Bruker AXS GmbH*. X‐ray radiation was generated by microfocus source *IμS* Mo (*λ*=0.71073 Å) by *Incoatec GmbH* with HELIOS mirror optics and a single‐hole collimator by *Bruker AXS GmbH*. For the data collection, the programs APEX 4 Suite (v.2021.4‐0) with the integrated programs SAINT (integration) and SADABS (adsorption correction) by *Bruker AXS GmbH* were used. The processing and finalization of the crystal structure was done with the program Olex2.^[2]^ The crystal structure was solved with the ShelXT^[3]^ structure solution program using Intrinsic Phasing and refined with the ShelXL refinement package using Least Squares minimization. The non‐hydrogen atoms were refined anisotropically. *U*_eq_ is defined as one third of the trace of the orthogonalized tensor *U*_ij_. For the hydrogen atoms the standard values of the SHELXL^[4]^ program were used with *U*_iso_(H)=−1.2 *U*_eq_(C) for CH_2_ and CH and with *U*_iso_(H)=−1.5 *U*_eq_(C) for CH_3_.

**Compound** (*rac*)‐**3**: To a stirred solution of *N*,*N*‐dimethyl(aminomethyl)ferrocene (**2**) (100 mg, 0.41 mmol, 1.0 equiv) and potassium‐*tert*‐butoxide (92 mg, 0.82 mmol, 2.0 eq) in THF (1 ml) at −80 °C *n*‐butyllithium (0.33 ml, 0.82 mmol, 2.0 equiv; 2.5 M in hexane) was added dropwise. The metalated solution was stored at −80 °C and after one week a crop of yellow block could be obtained, suitable for single crystal X‐ray analysis. The crystals were washed with *n*‐pentane (3×1 ml) and dried in the vacuum at low temperatures. Due to the weak THF‐metal bond the crystals were not weight stable at room temperature, but with a cooled sample the yield of 125 mg (61 %, 0.25 mmol) could be estimated. ^**1**^ **H NMR** (400.25 MHz, THF‐d_8_): *δ*=1.13 [s, 18H; OC(C*H*_3_)_3_], 1.76–1.79 [m, 16H; *β*‐C*H*_2_ (THF)], 2.07 [s, 12H; N(C*H*_3_)_2_], 3.23 [s, 4H; C_ips°_C*H*_2_N(CH_3_)_2_], 3.60–3.64 [m, 16H; *α*‐C*H*_2_ (THF)], 4.05–4.11 (m, 16H; CpH_*α*_ and CpH_*β*_’s) ppm. **{^1^H}^13^C NMR** (100.64 MHz, THF‐d_8_): *δ*=26.6 [8C; *β*‐*C*H_2_ (THF)], 30.8 [6C; OC(*C*H_3_)_3_], 45.3 [4C; N(*C*H_3_)_2_], 60.2 [2C; C*C*H_2_N(CH_3_)_2_], 68.4 [8C; *α*‐*C*H_2_ (THF)], 67.9 [1C; Cp‐*C*H], 68.1 [1C; Cp‐*C*H], 68.5 [1C; Cp‐*C*H], 69.3 [5C, Cp‐*C*H], 71.0 [1C, *C*K] ppm (no signal of [Cp*C*CH_2_N(CH_3_)_2_] was observed in the ^13^C NMR spectrum). **{^1^H}^7^Li NMR**(155.55 MHz, THF‐d_8_): *δ*=1.5 [*Li*OC(CH_3_)_3_] ppm.

**Compound** (*R*_p_)‐**3**: *N*,*N*‐dimethyl(aminomethyl)ferrocene (**2**) (1.00 g, 4.11 mmol, 1.0 equiv.) was dissolved in diethyl ether (5 ml) and allowed to cool to −80 °C. Subsequently, *iso*‐propyllithium (7.04 ml, 4.93 mmol, 1.2 equiv, 0.7 M in *n*‐pentane) and (*R*,*R*)‐TMCDA (1.40 g, 8.22 mmol, 2.0 equiv) was added and the red reaction solution was stirred for 1 h and stored at −80 °C. After three days, red blocks of compound (*S*_p_)‐**2 a** were formed, which were washed with cold *n*‐pentane (2×5 ml) and cold THF (5 ml). The crystals were then carefully dried in fine vacuum and taken up in fresh THF (5 ml) at −80 °C. Then potassium *tert*‐butoxide (461 mg, 4.11 mmol, 1.0 equiv) was added and the resulting red reaction solution was stirred for 1 h at a maximum of −50 °C. Storage at −80 °C gave compound (*R*_p_)‐**3** in the form of yellow plates with a yield of 46 % (955 mg, 1.89 mmol).

**Compound 8**: To (*rac)*‐**3** (252 mg, 0.50 mmol, 1.0 equiv) toluene (1.0 ml, 10 mmol, 20.0 equiv) was added at −80 °C in THF (2 ml). The red precipitate formed was again dissolved in THF (2 ml), stirred for 2 h and warmed up to room temperature. Subsequently the red solution was cooled to −80 °C and chloro(trimethyl)silane (0.2 ml, 1.58 mmol, 3.2 equiv) was added dropwise. After 2 h all volatiles of the colorless solution were removed in the vacuo and the crude product was analyzed by GC/EI‐MS and NMR spectroscopy. The silylated product **8** could be obtained in a yield of 96 % (79 mg, 0.48 mmol), calculated by the integral ratio of the ^1^H NMR spectrum. The silylated product of (*rac)*‐**3** was not detected. ^**1**^ **H NMR** (400.25 MHz, THF‐d_8_): *δ*=0.13 [s, 9H; Si(C*H*_3_)_3_], 1.32 [s, 2H; PhC*H*_2_Si(C*H*_3_)_3_], 7.24–7.34 (m, 5H, *H*_ar_) ppm. **GC/EI‐MS** (70 eV, *t_R_* =4.07 min): *m*/*z* (%)=164 (19) (M^+⋅^), 149 (11) [(M−CH_3_)^+^], 133 (1) [(M−CH_3_)_2_^+^], 91 (10) [(C_7_H_7_)^+^], 73 (100) [(C_3_H_9_Si)^+^].

**Compound 6**: Crystals of (*rac*)‐3 (434 mg, 0.86 mmol, 1.0 equiv) were dissolved in THF (2 ml) and *N,N*‐dimethylbenzylamine (0.12 ml, 0.86 mmol, 1 equiv) was added at ‐80 °C. Then, the mixture was stirred for 2 h, allowing the temperature to warm up to a maximum of −50 °C. Afterwards, the dark red reaction mixture was cooled to −80 °C and chloro(trimethyl)silane (0.27 ml, 2.15 mmol, 2.5 equiv) was added dropwise. After 2 h the volatiles were removed in the vacuo and the crude product was analyzed via GC/EI‐MS and NMR spectroscopy. The silylated product 6 could be obtained in a yield of >99 % (178 mg, 0.86 mmol), calculated by the integral ratio of the ^1^H NMR spectrum. The silylated product of (*rac*)‐3 was not detected. ^**1**^ **H** **NMR** (500.04 MHz, CDCl_3_) *δ*=−0.03 [s, 9H; Si(C*H*_3_)_3_], 2.28 [s, 6H; N(C*H*_3_)_2_], 2.65 (s, 1H; SiC*H*N), 7.12–7.24 (m, 5H; *H*_ar_) ppm. **{^1^H}^13^C** **NMR** (125.73 MHz, CDCl_3_) *δ*=−1.0 [3C; Si(*C*H_3_)_3_], 47.2 [2C; N(*C*H_3_)_2_], 67.6 (1C; Si*C*HN), 125.7 (1C; *C_para_*), 128.2 (4C; *C_ortho_*, *C_meta_*), 143.3 (1C; *C_ipso_*) ppm. **{^1^H}^29^Si** **NMR** (79.51 MHz, CDCl_3_) *δ*=0.67 (1Si; *Si*) ppm. **GC/EI‐MS** (70 eV, *t_R_* =7.29 min): *m/z* (%)=207 (12) (*M* ^+^), 192 (24) [(*M*−CH_3_)^+^],164 (2) [(C_10_H_16_Si)^+^], 135 (100) [(M−SiC_3_H_9_)^+^], 91 (26) [(C_7_H_8_)^+^], 77 (10) [(C_6_H_5_)^+^].

**Compound 4**: *N,N*‐dimethylbenzylamine (1) (0.53 ml, 3.60 mmol, 1 equiv) was dissolved in THF (2 ml) and cooled to −80 °C. Afterwards, potassium‐*tert*‐butoxide (3.60 ml, 3.60 mmol, 1 equiv, 1 M in THF) and *n*‐butyllithium (1.44 ml, 3.60 mmol, 1 equiv, 2.5 M in hexane) were added to give a dark red solution, which was allowed to warm up to −50 °C. It was layered with *n*‐pentane (1 ml) and stored at −80 °C. After two days red block‐shaped crystals of compound 5 formed, suitable for single crystal X‐ray analysis. The mother liquor was removed and the crystals were washed with cold *n*‐pentane (3×1 ml) until the solvent remained colorless. Subsequently, the crystals were carefully dried in the vacuum, but due to the weak THF metal bond decomposition of the crystals at room temperature occurred. At lower temperatures a yield of 82 % (937 mg, 2.95 mmol) could be estimated. The results of the NMR studies are in good agreement with the data in the literature.^[8]^

**X‐ray crystallography**: 2106893 [for (*rac*)‐**3**], 2106894 [for (*R*_p_)‐**3**], 2051722 (for **4**), 2106925 (for **5**) contain(s) the supplementary crystallographic data for this paper. These data are provided free of charge by the joint Cambridge Crystallographic Data Centre and Fachinformationszentrum Karlsruhe Access Structures service.

## Conflict of interest

The authors declare no conflict of interest.

1

## Supporting information

As a service to our authors and readers, this journal provides supporting information supplied by the authors. Such materials are peer reviewed and may be re‐organized for online delivery, but are not copy‐edited or typeset. Technical support issues arising from supporting information (other than missing files) should be addressed to the authors.

Supporting Information

## Data Availability

The data that support the findings of this study are available in the supplementary material of this article.
