# Supplementary material for: Towards Substrate–Reagent Interaction of Lochmann–Schlosser Bases in THF: Bridging THF Hides Potential Reaction Site of a Chiral Superbase
Source: Chemistry. 2022 Oct 17;28(69):e202202660. doi: 10.1002/chem.202202660 (PMC10092790; doi:10.1002/chem.202202660)
Supplement: Supplementary file 1 — Supporting Information [file CHEM-28-0-s001.pdf]

Chemistry–A European Journal

Supporting Information

Towards Substrate–Reagent Interaction of Lochmann–Schlosser Bases in THF: Bridging THF Hides Potential Reaction Site of a Chiral Superbase

Lukas Brieger, Tobias Schrimpf, Rebecca Scheel, Christian Unkelbach, and Carsten Strohmann*

1. General Remarks

All reactions with oxygen- and moisture-sensitive compounds were performed under an atmosphere of argon in dried solvents, which were distilled prior to use. All other solvents and commercially available reagents, including the NMR solvents, were used without further purification.

The NMR spectra were measured on a *Bruker Avance DRX-400* and on a *Bruker Avance DRX-500* NMR spectrometer. All NMR spectra were recorded at room temperature (ca. 22 °C). Chemical shifts (δ in ppm) are referred to tetramethylsilane (TMS), with the deuterium signal of the solvent serving as internal lock and the residual solvent signal as additional reference [^{1}H-NMR: $\delta(C_6D_5H)$ = 7.16, $\delta(C_4D_7\mathit{H}O)$ = 1.73; 3.58, $\delta(C\mathit{H}Cl_3)$ = 7.26; ^{13}C-NMR: $\delta(\mathit{C}_6D_6)$ = 128.4, $\delta(\mathit{C}_4D_8O)$ = 25.3; 67.2, $\delta(\mathit{C}DCl_3)$ = 77.0]. The ^{29}Si-NMR experiments are referred to TMS as external standard and measured via the INEPT puls sequence. For the assignment of the multiplicities the following abbreviations were used: s = singlet, d = doublet, t = triplet, m = multiplet, br = broad signal. Aromatic carbon and hydrogen atoms were assigned as follows: i = ipso, o = ortho, m = meta, p = para.

GC/EI-MS analyses were obtained using an Agilent 7890B GC system (column: Agilent HP-5MS, 30 m, 0.25 mm, 0.25 µm) with an Agilent 5977A Mass Selective Detector.

Suitable crystals of compound (*rac*)-**3**, (R_p)-**3**, **4** and **5** were covered with an inert oil (perfluoroalkylether) at –80 °C using the *X-TEMP 2*[1] device in combination with a *SMZ1279* stereomicroscope from *Nikon Metrology GmbH* and mounted on a *MicroMount* from *MiTeGen*. Crystal structure determination was accomplished on a *Bruker D8 Venture* four-circle diffractometer using a *PHOTON II CPAD* detector by *Bruker AXS GmbH*. X-ray radiation was generated by microfocus source *IµS* Mo (λ = 0.71073 Å) by *Incoatec GmbH* with HELIOS mirror optics and a single-hole collimator by *Bruker AXS GmbH*. For the data collection, the programs APEX 4 Suite (v.2021.4-0) with the integrated programs SAINT (integration) and SADABS (adsorption correction) by *Bruker AXS GmbH* were used. The processing and finalization of the crystal structure was done with the program Olex2.[2] The crystal structure was solved with the ShelXT[3] structure solution program using Intrinsic Phasing and refined with the ShelXL refinement package using Least Squares minimization. The non-hydrogen atoms were refined anisotropically. U_{eq} is defined as one third of the trace of the orthogonalized tensor U_{ij}. For the hydrogen atoms the standard values of the SHELXL[4] program were used with $U_{iso}(H) = -1.2\ U_{eq}(C)$ for CH_2 and CH and with $U_{iso}(H) = -1.5\ U_{eq}(C)$ for CH_3.

2. Experimental Procedures

2.1 Synthesis of Benzyl(trimethyl)silan (8) with (*rac*)-3 by deprotonation of toluene (7)

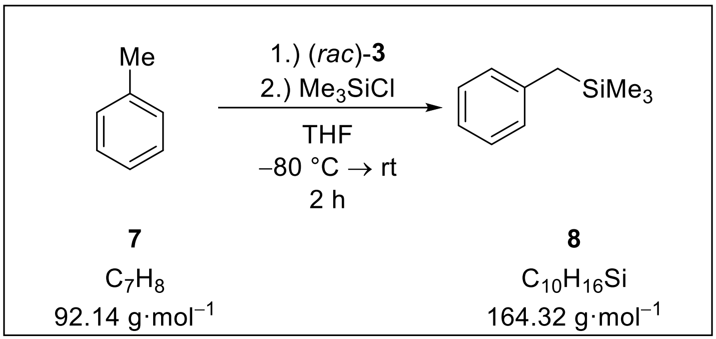

To (*rac)*-**3** (252 mg, 0.50 mmol, 1.0 eq.) toluene (1.0 ml, 10 mmol, 20.0 eq.) was added at –80 °C in THF (2 ml). The red precipitate formed was again dissolved in THF (2 ml), stirred for 2 h and warmed up to room temperature. Subsequently the red solution was cooled to –80 °C and chloro(trimethyl)silane (0.2 ml, 1.58 mmol, 3.2 eq.) was added dropwise. After 2 h all volatiles of the colorless solution were removed in the vacuo and the crude product was analyzed by GC/EI-MS and NMR spectroscopy. The silylated product **8** could be obtained in a yield of 96% (79 mg, 0.48 mmol), calculated by the integral ratio of the ^{1}H-NMR spectrum. The silylated product of (*rac)*-**3** was not detected.

^{1}H-NMR (400.25 MHz, THF-d$_8$): δ = 0.13 [s, 9H; Si(CH_3)$_3$], 1.32 [s, 2H; PhCH_2Si(CH_3)$_3$], 7.24–7.34 (m, 5H, H_{ar}) ppm.

GC/EI-MS (70 eV, t_R = 4.07 min): *m*/*z* (%) = 164 (19) ($M^{+\cdot}$), 149 (11) [(M-CH$_3$)$^+$], 133 (1) [(M-CH$_3$)$_2$$^+$], 91 (10) [($C_7H_7$)$^+$], 73 (100) [($C_3H_9Si$)$^+$].

2.2 Synthesis of *N*,*N*-dimethylbenzyl(trimethylsilyl)amine (6) with (*rac*)-3 by deprotonation of *N*,*N*-dimethylbenzylamine (1)

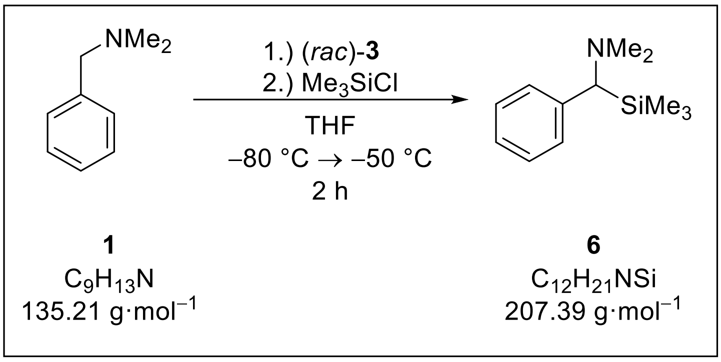

Crystals of (*rac*)-**3** (434 mg, 0.86 mmol, 1.0 eq.) were dissolved in THF (2 ml) and *N,N*-dimethylbenzylamine (0.12 ml, 0.86 mmol, 1 eq.) was added at –80 °C. Then, the mixture was stirred for 2 hours, allowing the temperature to warm up to a maximum of –50 °C. Afterwards, the dark red reaction mixture was cooled to –80 °C and chloro(trimethyl)silane (0.27 ml, 2.15 mmol, 2.5 eq.) was added dropwise. After 2 hours the volatiles were removed in the vacuo and the crude product was analyzed via GC/EI-MS and NMR spectroscopy. The silylated

product **6** could be obtained in a yield of >99% (178 mg, 0.86 mmol), calculated by the integral ratio of the ^{1}H-NMR spectrum. The silylated product of (*rac*)-**3** was not detected.

^{1}H NMR (500.04 MHz, $CDCl_3$) δ = –0.03 [s, 9H; Si(C*H*$_3$)$_3$], 2.28 [s, 6H; N(C*H*$_3$)$_2$], 2.65 (s, 1H; SiC*H*N), 7.12 – 7.24 (m, 5H; H_{ar}) ppm.

{^{1}H}^{13}C NMR (125.73 MHz, $CDCl_3$) δ = –1.0 [3C; Si(*C*H$_3$)$_3$], 47.2 [2C; N(*C*H$_3$)$_2$], 67.6 (1C; Si*C*HN), 125.7 (1C; C_{para}), 128.2 (4C; C_{ortho}, C_{meta}), 143.3 (1C; C_{ipso}) ppm.

{^{1}H}^{29}Si NMR (79.51 MHz, $CDCl_3$) δ = 0.67 (1Si; *Si*) ppm.

GC/EI-MS (70 eV, t_R = 7.29 min): *m/z* (%) = 207 (12) (M^+), 192 (24) [(M–CH_3)$^+$],164 (2) [($C_{10}H_{16}Si$)$^+$], 135 (100) [(M–SiC_3H_9)$^+$], 91 (26) [(C_7H_8)$^+$], 77 (10) [(C_6H_5)$^+$].

2.3 Synthesis of *N*,*N*-dimethylbenzyl(trimethylsilyl)amine (6) with (R_p)-3 by deprotonation of *N*,*N*-dimethylbenzylamine (1)

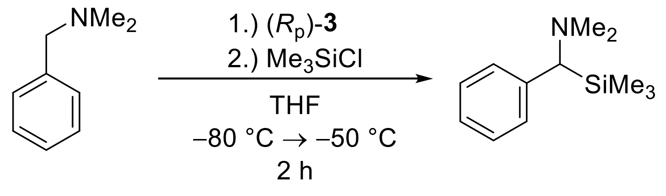

1
$C_9H_{13}N$
135.21 g·mol^{-1}

6
$C_{12}H_{21}NSi$
207.39 g·mol^{-1}

Crystals of (R_p)-**3** (813 mg, 1.61 mmol, 1.0 eq.) were dissolved in THF (2 ml) and *N,N*-dimethylbenzylamine (0.23 ml, 1.61 mmol, 1 eq.) was added at –80 °C. Then, the mixture was stirred for 2 hours, allowing the temperature to warm up to a maximum of –50 °C. Afterwards, the dark red reaction mixture was cooled to –80 °C and chloro(trimethyl)silane (0.51 ml, 4.03 mmol, 2.5 eq.) was added dropwise. After 2 hours the volatiles were removed in the vacuo and the crude product was analyzed via GC/EI-MS and NMR spectroscopy. The silylated product **6** could be obtained in a quantitative yield of >99% (334 mg, 1.61 mmol), calculated by the integral ratio of the ^{1}H-NMR spectrum. The silylated product of (R_p)-**3** was not detected. To to determine the enantiomeric ratio (*R*)-mandelic acid was added. The *e.r.* is 1:1.

(*R*)-**6**

^{1}H NMR (500.04 MHz, $CDCl_3$) δ = 0.05 [s, 9H; Si(C*H*$_3$)$_3$], 2.64 [s, 6H; N(C*H*$_3$)$_2$], 3.44 (s, 1H; SiC*H*N), 7.21-7.35 (m, 5H; $C_{Ar}H$) ppm.

{^{1}H}^{13}C NMR (125.73 MHz, $CDCl_3$) δ = –1.2 [3C; Si(*C*H$_3$)$_3$], 44.2 [2C; N(*C*H$_3$)$_2$], 67.0 (1H; Si-*C*H-N), 73.7 (1C, Ph*C*HOH), 129.3 (4C; C_{ortho}, C_{meta}, C_{para}), 134.1 (1C, C_{ipso}) ppm.

{^{1}H}^{29}Si NMR (79.51 MHz, $CDCl_3$) δ = 3.04 (1Si; *Si*) ppm.

(*S*)-**6**

^{1}H NMR (500.04 MHz, $CDCl_3$) δ = 0.10 [s, 9H; Si(C*H*$_3$)$_3$], 2.65 [s, 6H; N(C*H*$_3$)$_2$], 3.42 (s, 1H; Si-C*H*-N), 7.21 – 7.35 (m, 5H; C_{Ar}-*H*) ppm.

{^{1}H}^{13}C NMR (125.73 MHz, $CDCl_3$) δ = –1.3 [3C; Si(CH_3)$_3$], 44.2 [2C; N(*C*H$_3$)$_2$], 67.0 (1H; Si-*C*H-N), 73.7 (1C, Ph*C*HOH), 129.3 (5C; *C*$_{ortho}$, *C*$_{meta}$, *C*$_{para}$), 134.1 (1C, *C*$_{ipso}$) ppm.

{^{1}H}^{29}Si NMR (79.51 MHz, $CDCl_3$) δ = 3.04 (1Si; *Si*) ppm.

GC/EI-MS (70 eV, t_R = 7.29 min): *m/z* (%) = 207 (12) (M^+), 192 (24) [(M–CH_3)$^+$],164 (2) [($C_{10}H_{16}Si$)$^+$], 135 (100) [(M–SiC_3H_9)$^+$], 91 (26) [(C_7H_8)$^+$], 77 (10) [(C_6H_5)$^+$].

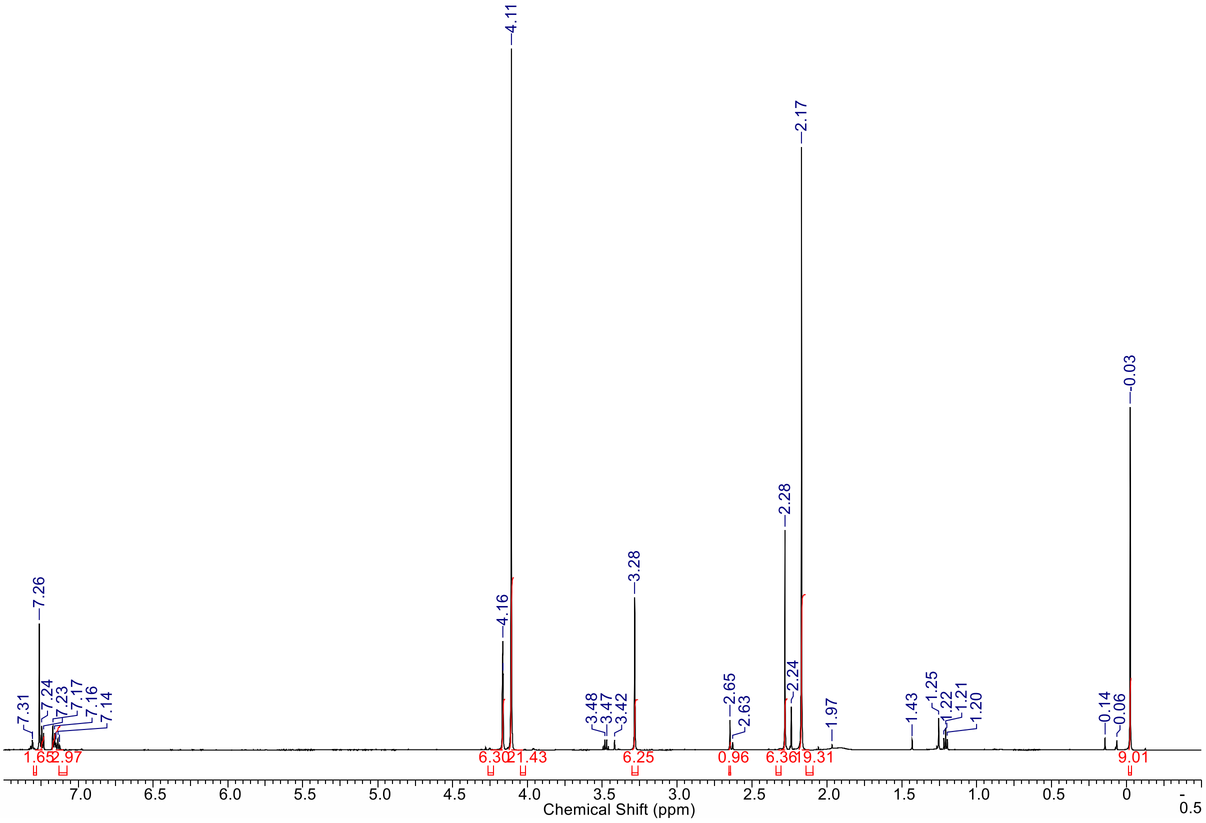

Figure S1 ^{1}H-NMR of **6**.

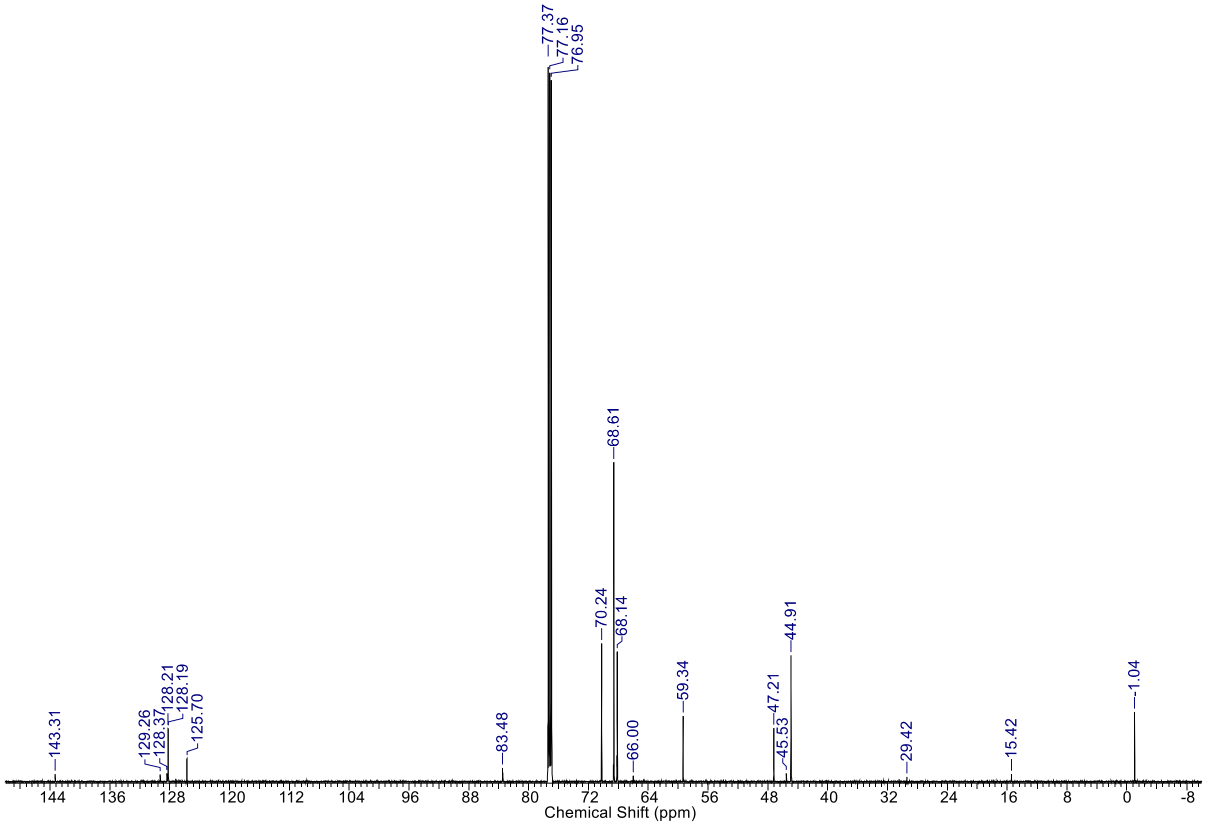

Figure S2 {^{1}H}^{13}C-NMR of **6**.

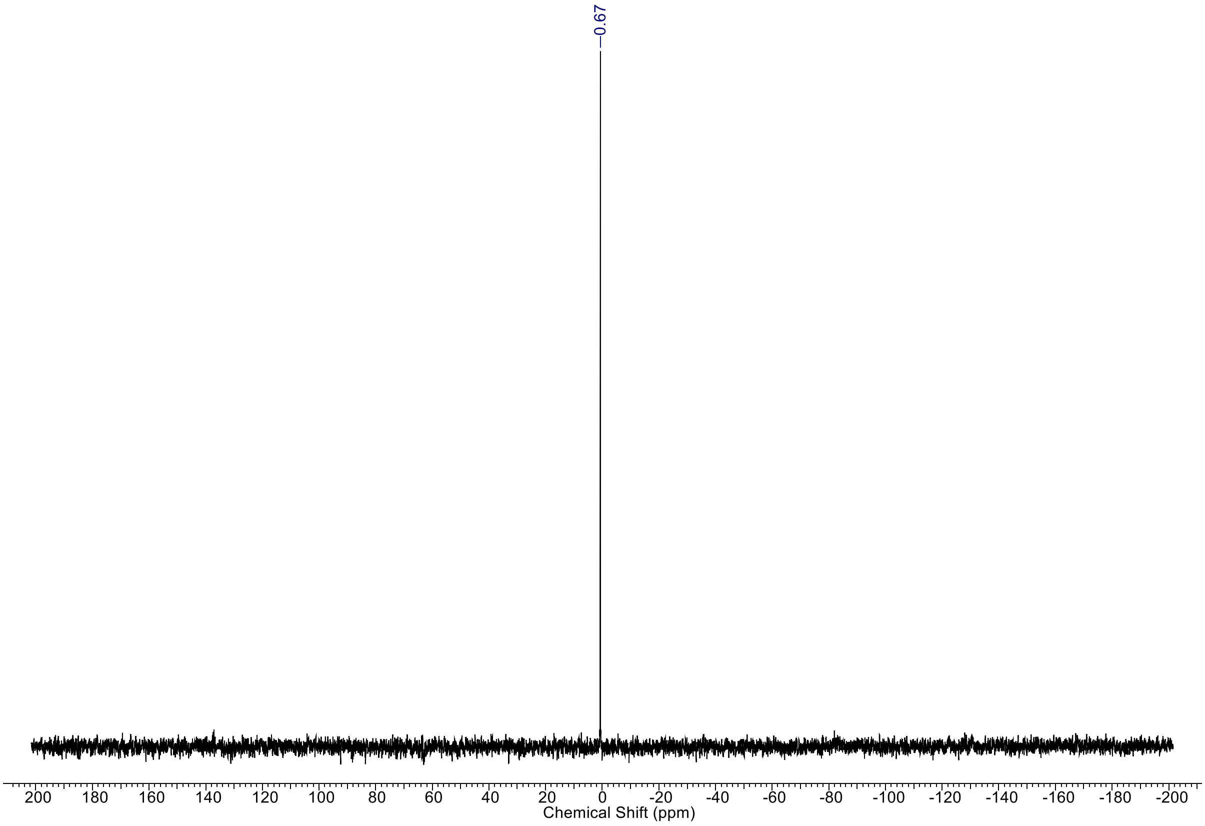

Figure S3 ^{29}Si-NMR spectrum of **6**.

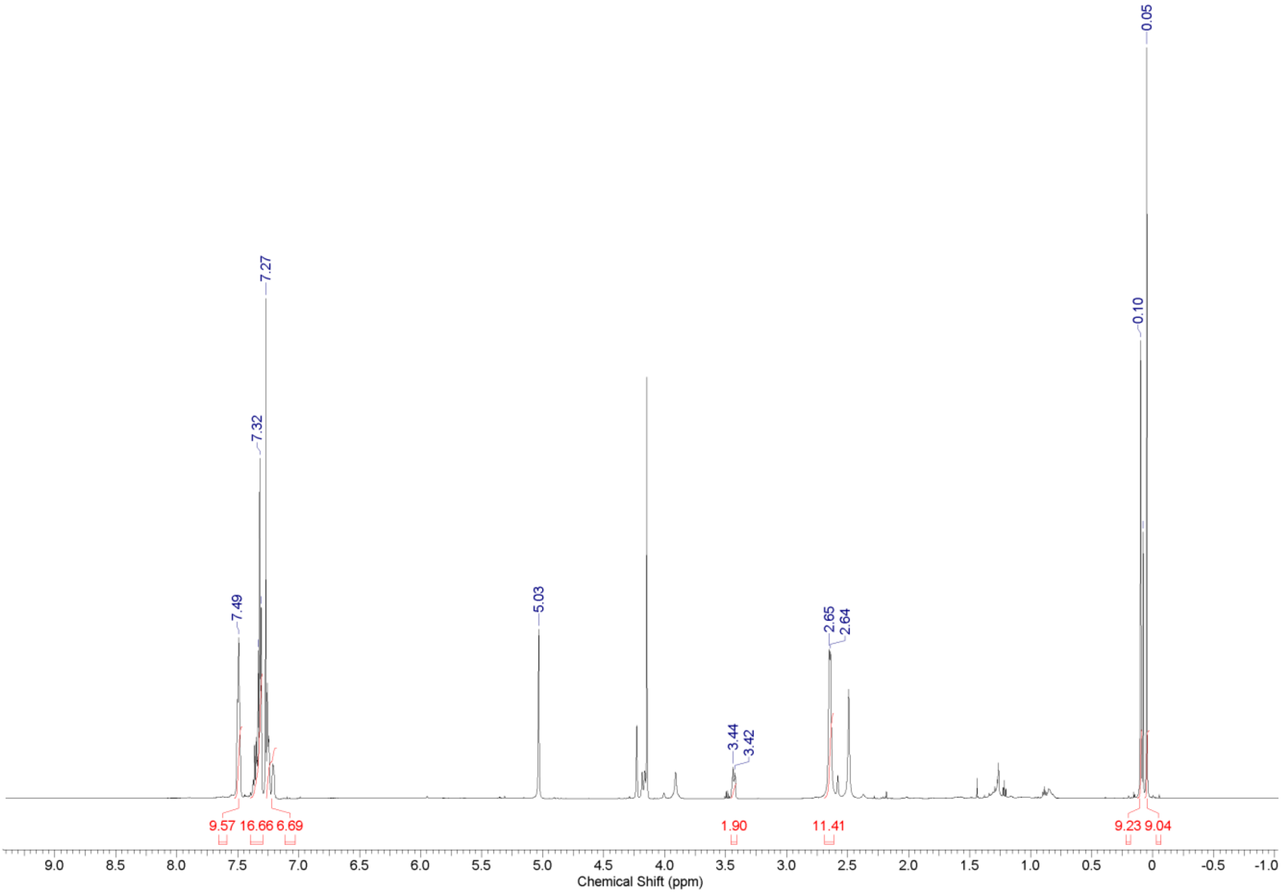

Figure S4 ^{1}H-NMR of **6** with (*R*)-mandelic acid.

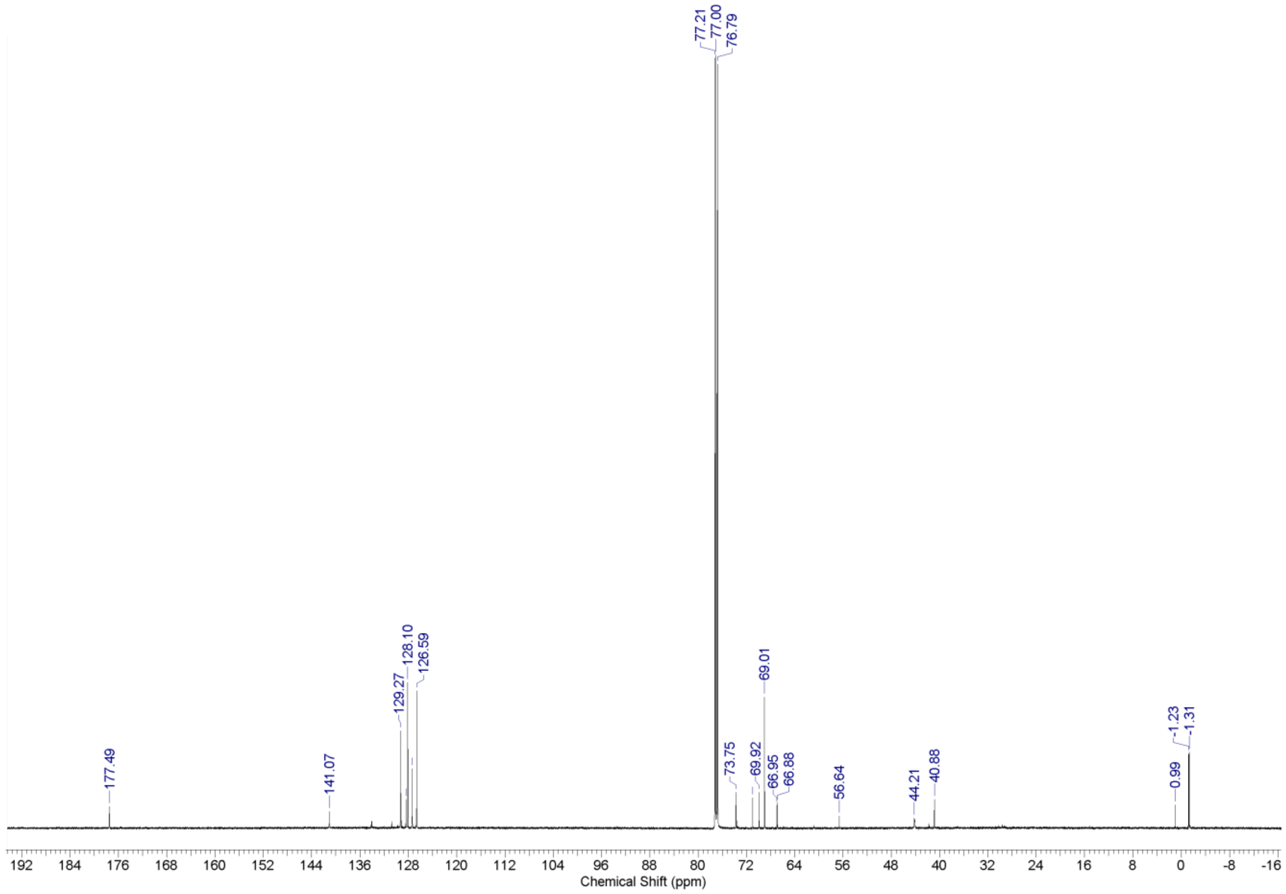

Figure S5 ^{13}C-NMR of **6** with (*R*)-mandelic acid.

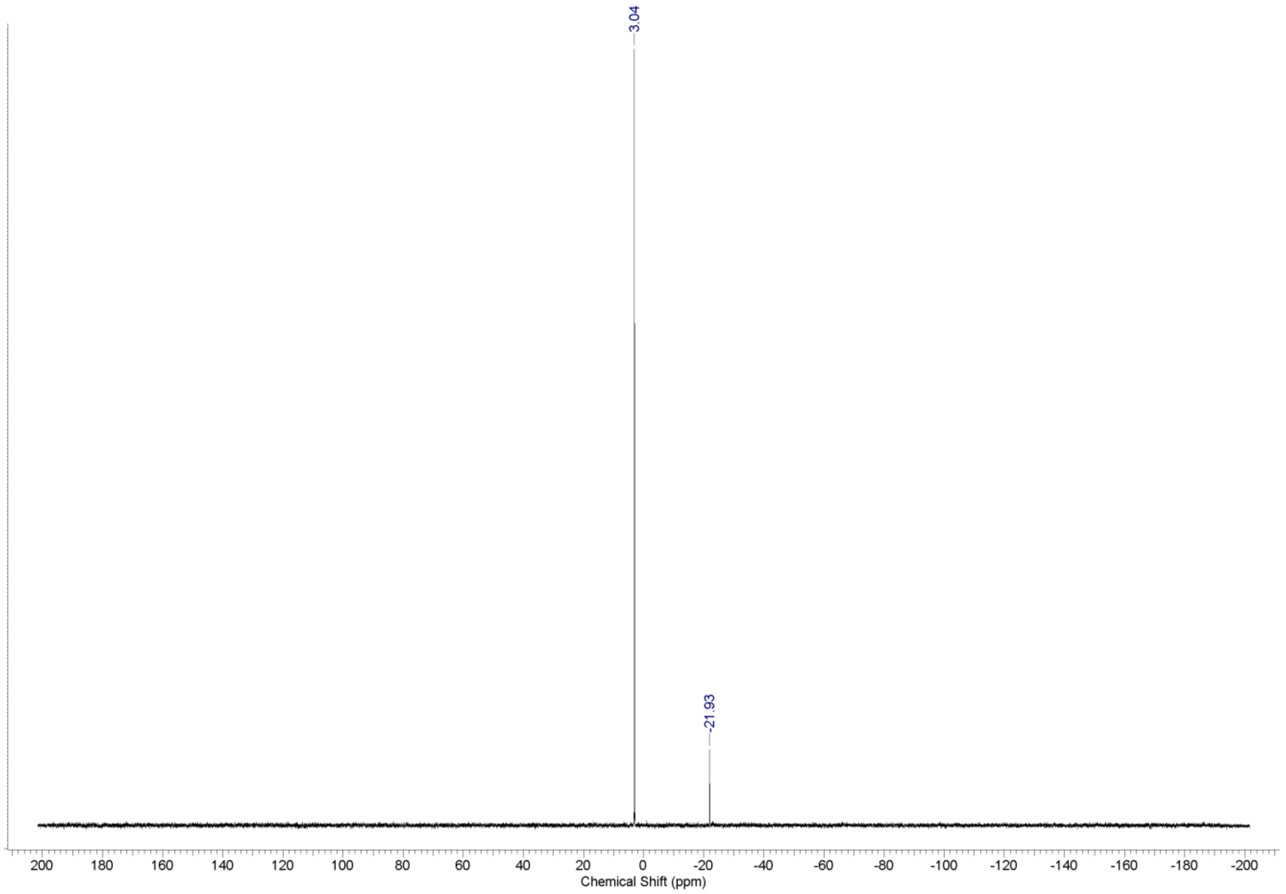

Figure S6 ^{29}Si-NMR of **6** with *R*)-mandelic acid.

3. Single crystal X-ray diffractional analysis

Crystallographic data for the structures of (*rac*)-**3**, (R_p)-**3**, **4** and **5** have been deposited with the Cambridge Crystallographic Data Centre as supplementary publication numbers (Table S1). Copy of these data can be obtained, free of charge, on application to CCDC, 12 Union Road, Cambridge CB2 IEZ, UK, fax: 144-(0)1223-336033 or e-mail: deposit@ccdc.cam.ac.uk.

Table S1 CCDC deposition numbers for the crystallized compounds.

Compound	CCDC deposition number
(*rac*)-**3**	2106893
(R_p)-**3**	2106894
**4**	2051722
**5**	2106925

3.1 Synthesis of (*rac*)-3

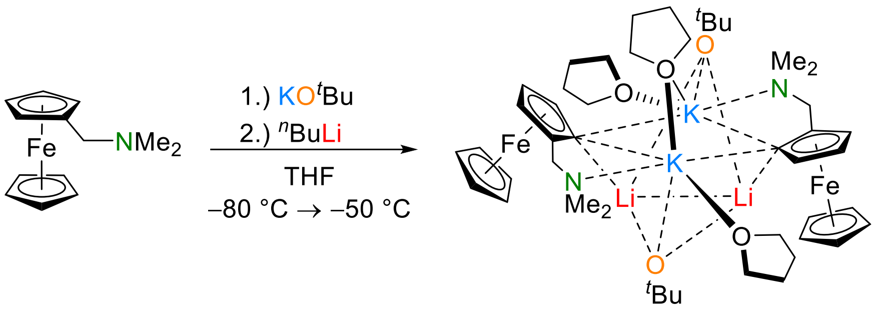

2
$C_{13}H_{17}FeN$
243.13 g·mol^{-1}

(*rac*)-**3**
$C_{50}H_{82}Fe_2K_2Li_2N_2O_6$
1010.95 g·mol^{-1}

To a stirred solution of *N*,*N*-dimethyl(aminomethyl)ferrocene (**2**) (100 mg, 0.41 mmol, 1.0 eq.) and potassium-*tert*-butoxide (92 mg, 0.82 mmol, 2.0 eq) in THF (1 ml) at –80 °C *n*-butyllithium (0.33 ml, 0.82 mmol, 2.0 eq.; 2.5 M in hexane) was added dropwise. The metalated solution was stored at –80 °C and after one week a crop of yellow block could be obtained, suitable for single crystal X-ray analysis. The crystals were washed with *n*-pentane (3 x 1 ml) and dried in the vacuum at low temperatures. Due to the weak THF-metal bond the crystals were not weight stable at room temperature, but with a cooled sample the yield of 125 mg (61%, 0.25 mmol) could be estimated.

3.2 Synthesis of (R_p)-3

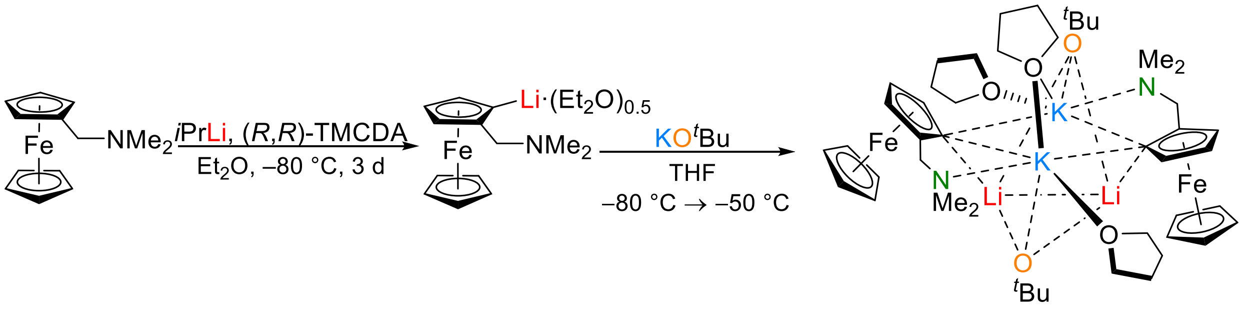

2
$C_{13}H_{17}FeN$
243.13 g·mol^{-1}

(S_p)-**2a**
$C_{12}H_{17}FeNLi$
243.13 g·mol^{-1}

(R_p)-**3**
$C_{50}H_{82}Fe_2K_2Li_2N_2O_6$
1010.95 g·mol^{-1}

N,*N*-dimethyl(aminomethyl)ferrocene (**2**) (1.00 g, 4.11 mmol, 1.0 equiv.) was dissolved in diethyl ether (5 ml) and allowed to cool to –80 °C. Subsequently, *iso*-propyllithium (7.04 ml, 4.93 mmol, 1.2 eq., 0.7 M in *n*-pentane) and (*R*,*R*)-TMCDA (1.40 g, 8.22 mmol, 2.0 eq.) was added and the red reaction solution was stirred for 1 h and stored at –80 °C. After three days, red blocks of compound (S_p)-**2a** were formed, which were washed with cold *n*-pentane (2 x 5 ml) and cold THF (5 ml). The crystals were then carefully dried in fine vacuum and taken up in fresh THF (5 ml) at –80 °C. Then potassium *tert*-butoxide (461 mg, 4.11 mmol, 1.0 eq.) was added and the resulting red reaction solution was stirred for 1 h at a maximum of –50 °C. Storage at –80 °C gave compound (R_p)-**3** in the form of yellow plates with a yield of 46% (955 mg, 1.89 mmol).

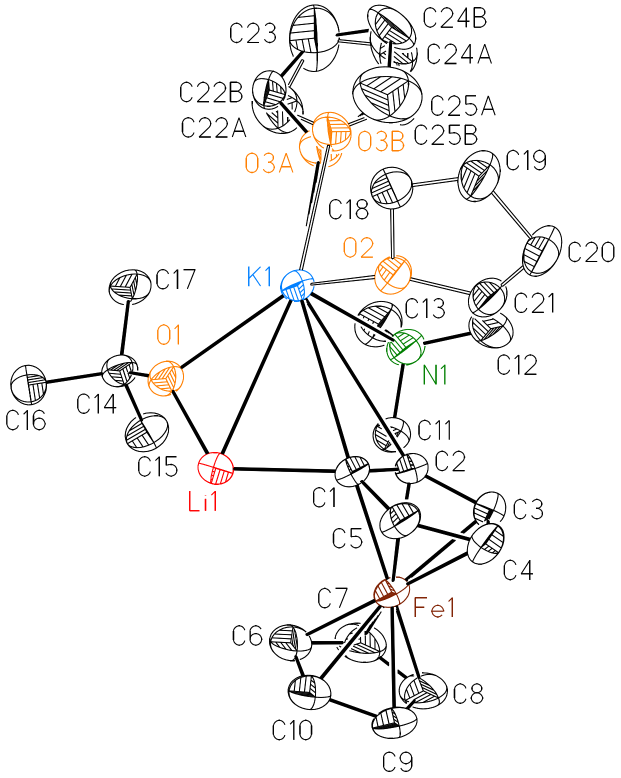

(*rac*)-**3**

Figure S7 Ortep plot[5] of the asymmetric unit of (*rac*)-**3** in the crystal with displacement ellipsoids drawn at the 50% probability level. Hydrogen atoms and disorders are omitted for clarity. Selected bond lengths [Å] and angles [°]: K1–O1 2.711(6), K1–O5 2.632(4), K1–C1 3.199(6), K2–C1 3.116(6), K1–C14 3.114(6), K2–C14 3.204(6) K1–Li1 3.256(10), K1–Li2 3.176(9), K1–N2 2.852(6), Li1–O4 1.887(10), Li2–O4 1.938(10); C14–K1–C1 103.3(2), K1–C1–K2 72.6(1), O1–K1–K2 142.5(2), O1–K1–N1 82.5(2), Li1–K1–K2 54.0(2).

^{1}H-NMR (400.25 MHz, THF-d$_8$): δ = 1.13 [s, 18H; OC(C*H*$_3$)$_3$], 1.76–1.79 [m, 16H; β-C*H*$_2$ (THF)], 2.07 [s, 12H; N(C*H*$_3$)$_2$], 3.23 [s, 4H; C$_{ipso}$C*H*$_2$N(CH$_3$)$_2$], 3.60–3.64 [m, 16H; α-C*H*$_2$ (THF)], 4.05–4.11 (m, 16H; CpH$_\alpha$ and CpH$_\beta$'s) ppm.

{^{1}H}^{13}C-NMR (100.64 MHz, THF-d$_8$): δ = 26.6 [8C; β-CH$_2$ (THF)], 30.8 [6C; OC(*C*H$_3$)$_3$], 45.3 [4C; N(*C*H$_3$)$_2$], 60.2 [2C; C*C*H$_2$N(CH$_3$)$_2$], 68.4 [8C; α-*C*H$_2$ (THF)], 67.9 [1C; Cp-*C*H], 68.1 [1C; Cp-*C*H], 68.5 [1C; Cp-*C*H], 69.3 [5C, Cp-*C*H], 71.0 [1C, *C*K] ppm.*

{^{1}H}^{7}Li-NMR (155.55 MHz, THF-d$_8$): δ = 1.5 [*Li*OC(CH$_3$)$_3$] ppm.

*no signal of [Cp*C*CH$_2$N(CH$_3$)$_2$] was observed in the ^{13}C-NMR spectrum.

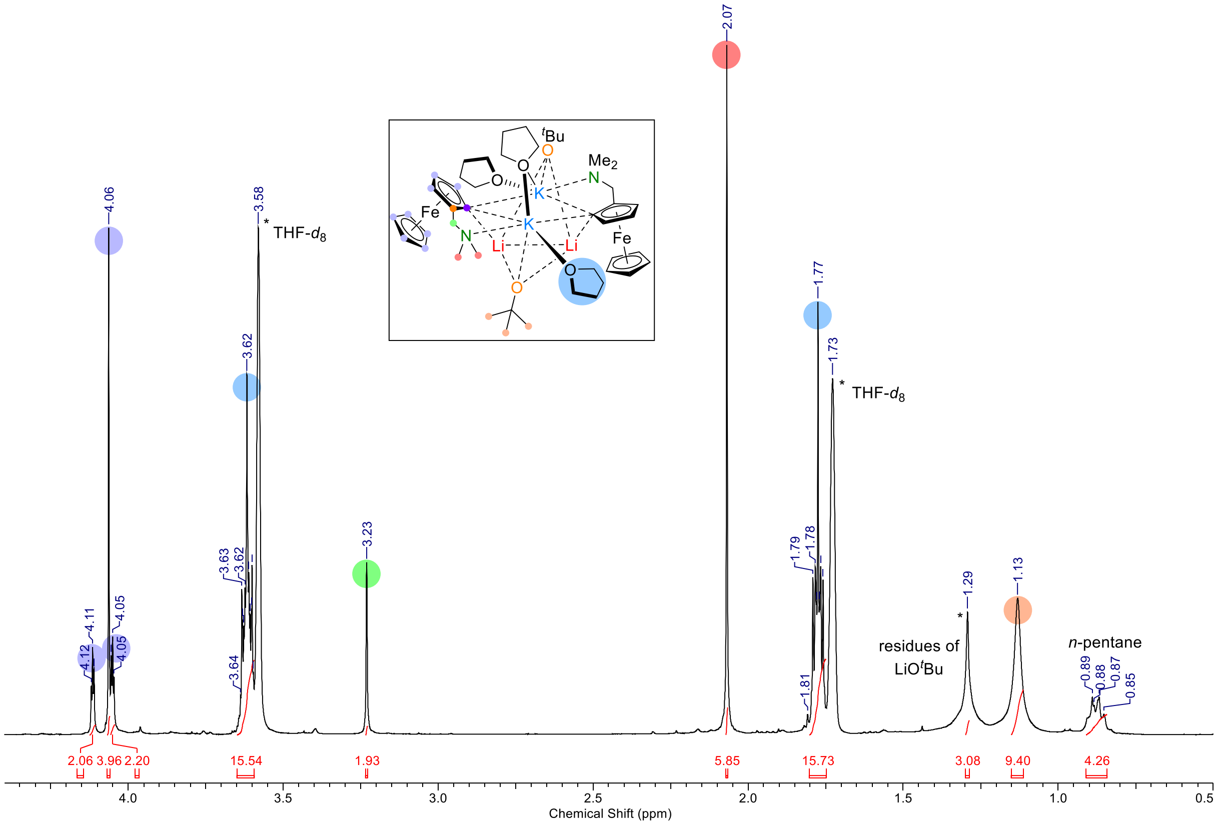

Figure S8 ^{1}H-NMR spectrum of (*rac*)-**3**.

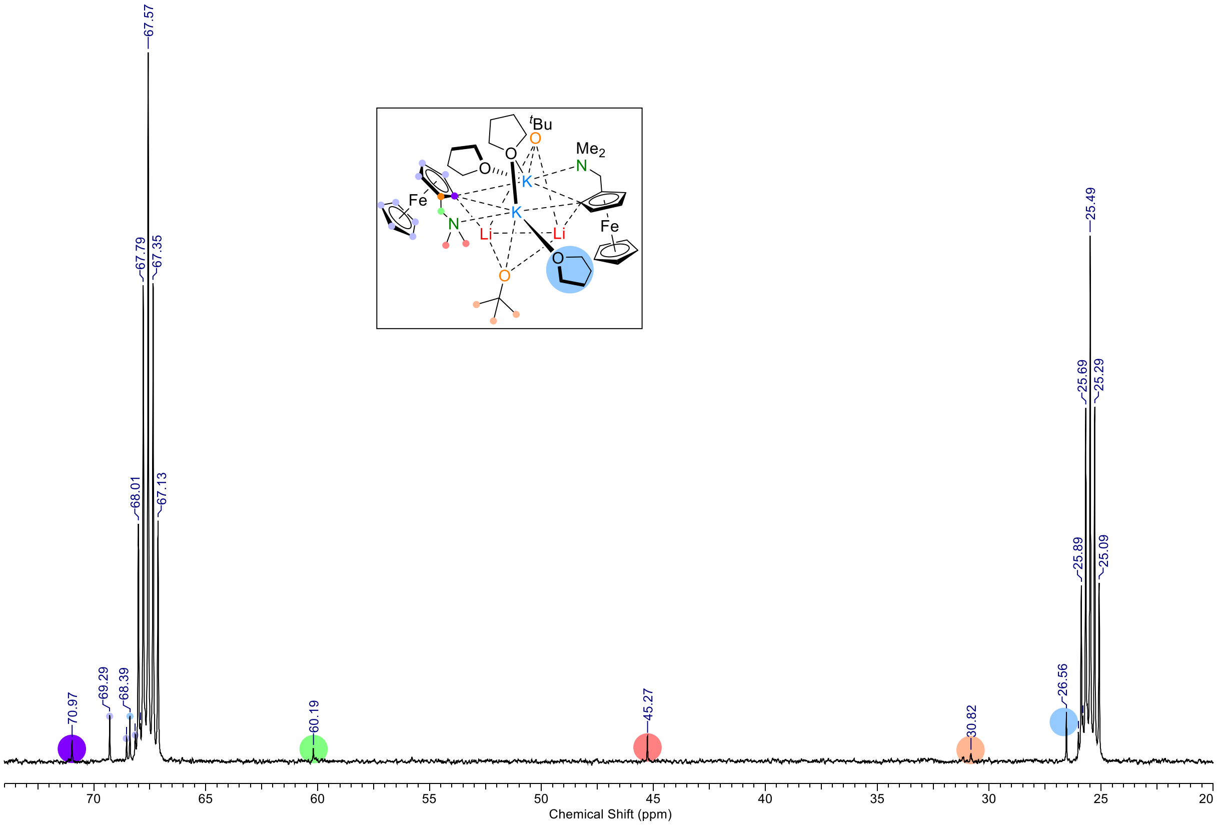

Figure S9 ^{13}C-NMR spectrum of (*rac*)-**3**.

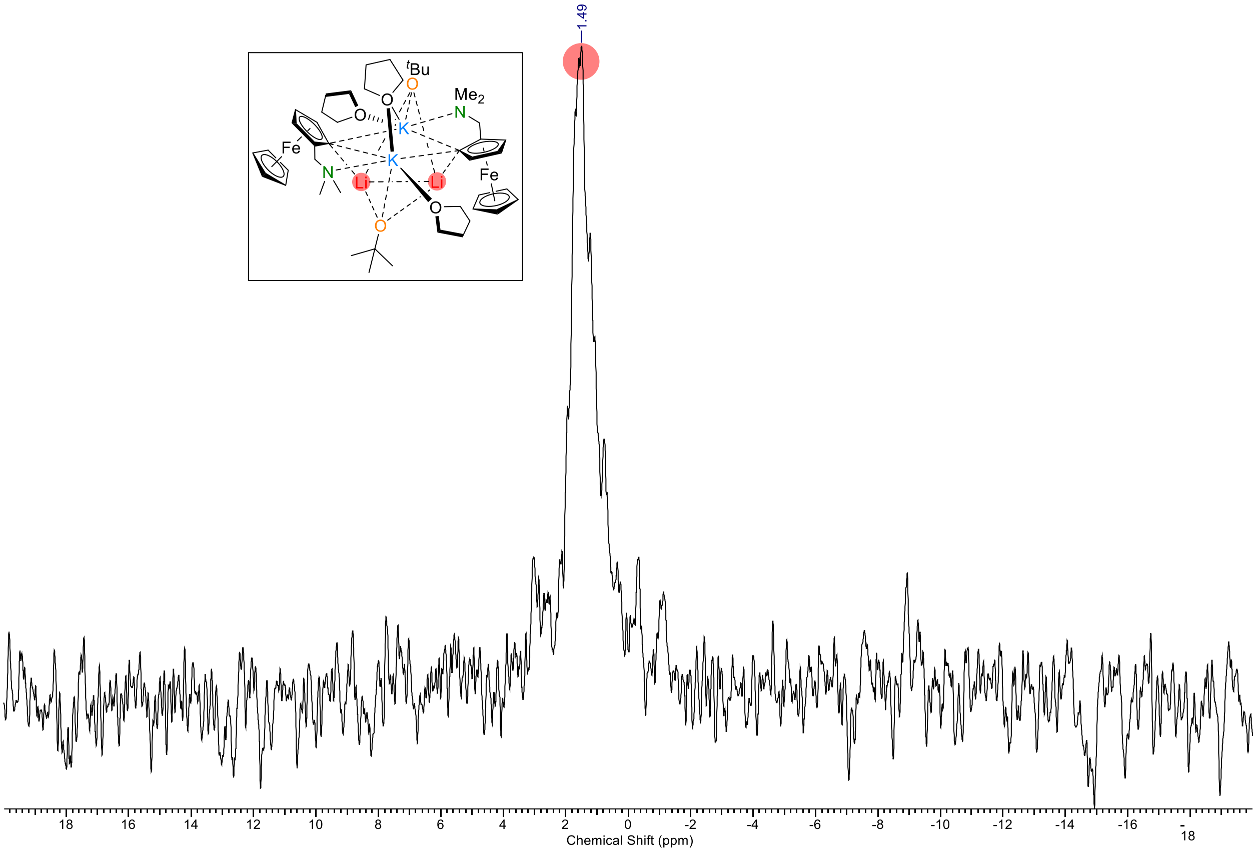

Figure S10 ^{7}Li-NMR spectrum of (*rac*)-**3**.

3.3 Synthesis of 4

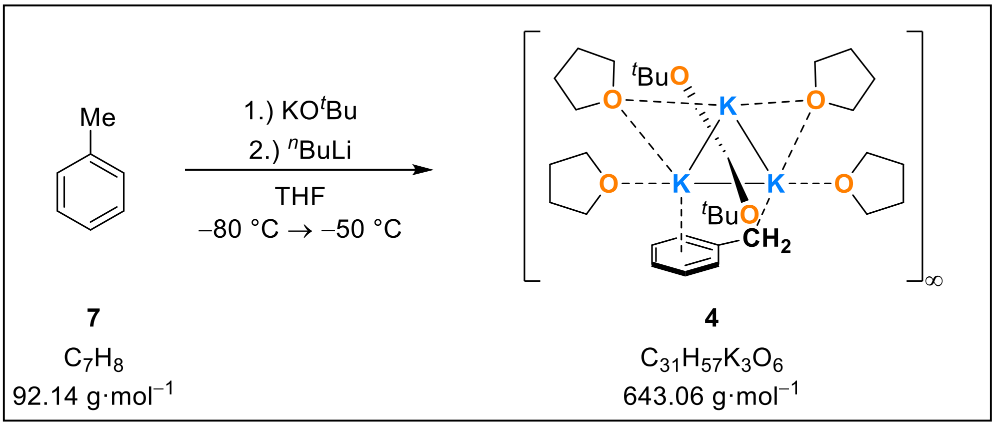

To a stirred solution of toluene (92 mg, 1.0 mmol, 1.0 eq.) and potassium *tert*-butoxide (336 mg, 3.0 mmol, 3.0 eq.) at –80 °C in THF *n*-butyllithium (0.44 ml, 1.1 mmol, 1.1 eq., 2.5 M in hexane) was added. The orange solution was stored at –80 °C. After two days a crop of yellow plates of compound **4** formed, suitable for single crystal X-ray analysis. Between the yellow plates we obtained orange red needles of the benzylpotassium THF solvate, which was already investigated by our group, indicating that the compound does not crystallize selectively.[6] Therefore, we were neither able to determine a yield nor pure NMR spectra of **4**. Hence, we only show the synthesis of the compound here in the ESI and do not elaborate further on the structure of this compound in the manuscript.

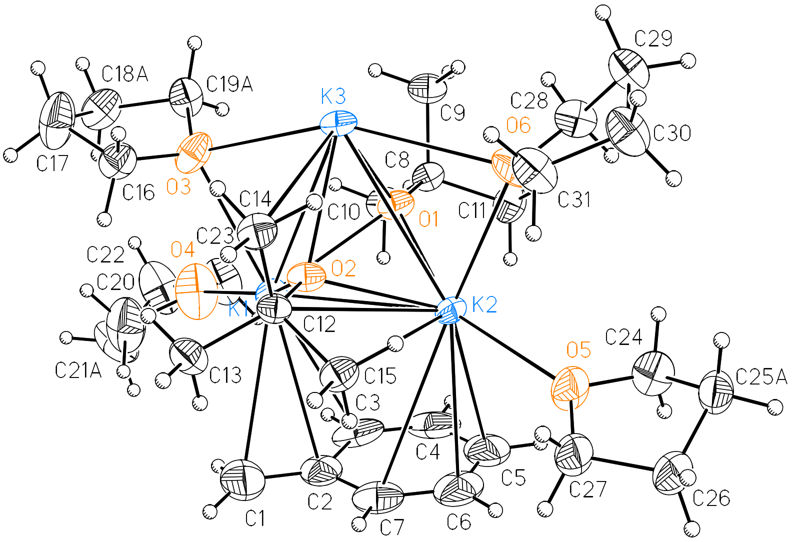

4

Figure S 11 Ortep plot[5] of the asymmetric unit of **4** in the crystal, with displacement ellipsoids drawn at the 50% probability level. Numbering scheme of hydrogen atoms and disorders are omitted for clarity. Selected bond lengths [Å] and angles [°]: K1–K2 3.403(1), K1–K3 3.571(1), K2–K3 3.562(1), K1–O1 2.619(2), K1–O2 2.602(2), K1–O3 2.746(2), K1–O4 2.812(2), K2–O5 2.760(2), K2–O6 2.802(2), K3–O3 2.893(2), K3–O6 3.055(2), K1–C1 3.202(4), K1–C2 3.139(3), K1–C3 3.169(3), K2–C5 3.250(4), K2–C6 3.117(3), K2–C7 3.417(3), K3–C2' 3.236(3), K3–C3' 3.225(3), K3–C4' 3.232(3), K3–C5' 3.250(4), K3–C6' 3.188(3), K3–C7' 3.183(3), C1–C2 1.359(5), C2–C3 1.460(5), C2–C7 1.440(5), C3–C4 1.353(6), C4–C5 1.389(7), C5–C6 1.399(6), C6–C7 1.372(6); C1–C2–C3 124.9(4), C1–C2–C7 123.3(4), C3–C2–C7 111.8(3), C4–C3–C2 122.7(4), C3–C4–C5 123.6(3), C4–C5–C6 116.4(4), C5–C6–C7 121.4(4), C6–C7–C2 124.1(3).

We were delighted to find that the composition of **4** was [$(PhCH_2K)(^tBuOK)_2(THF)_4$] and contained both benzylpotassium and potassium-*tert*-butoxide. The central motif of this solid-state structure comprises a benzylpotassium moiety connected with two potassium-*tert*-butoxides. The potassium centers form a triangle whose faces are capped by the alkoxide anions. Each potassium center has two contacts to the alkoxide anions, two contacts to coordinating THF molecules and *π*-type interactions with one benzyl anion saturating the coordination sphere. The metal center K1 is located above one half of the benzyl moiety with a η^3-contact to the benzyl-, *ipso*- and *ortho*-carbon center (Figure 6). Potassium center K2 is located above the other half of the benzyl moiety with a η^3 to the *para*-, *meta*- and the other *ortho*-carbon center. K3 is located below the arene plane of the next molecule with a η^6-contact to the benzyl anion moiety. The charge of the anion is delocalized, which is supported by the planarized carbanion [$\sum°_{(C1)} = 358.8°$] and the shortened benzylic-*ipso*-carbon bond with 1.360(5) Å (C1–C2), which is among the shortest recorded for benzyl metal compounds. This is also outlined by the position of the benzylic carbon center within the arene plane, which is hardly bent with 0.022(7) Å. In accordance, the *ipso*-*ortho*-carbon bonds are elongated [C2–C3 1.461(5) Å, C2–C7 1.440(5) Å] and the *ortho*-*meta*-carbon bonds are shortened [C3–C4 1.353(6) Å, C6–C7 1.370(6) Å]. Lochmann already described the formation of the benzylpotassium alkoxide adduct [$PhCH_2K(^tPeOK)_n]_m$[7] adding an excess of potassium *tert*-pentoxide to already formed benzylpotassium. After hydrolysis $PhCH_3$ and ^tPeOH in a ratio of 1:2 were found. These results are in good agreement with our observed intermediate [$(PhCH_2K)(^tBuOK)_2(THF)_4]_\infty$ (**4**). It seems there is no big difference using *tert*-butoxide instead of *tert*-pentoxide in terms of the stoichiometric ratio of the

adduct, but a different molecular structure can be considered as we used THF as additional Lewis base. Unfortunately, this compound does not crystallize selectively, so a solution phase NMR analysis was not possible.

3.4 Synthesis of 5

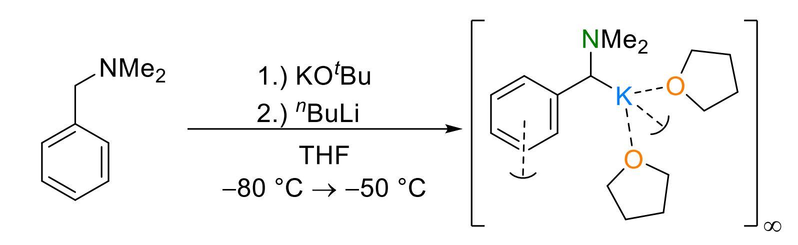

1
$C_9H_{13}N$
135.21 g·mol^{-1}

5
$C_{17}H_{28}KNO_2$
317.50 g·mol^{-1}

N,N-dimethylbenzylamine (**1**) (0.53 ml, 3.60 mmol, 1 eq.) was dissolved in THF (2 ml) and cooled to –80 °C. Afterwards, potassium-*tert*-butoxide (3.60 ml, 3.60 mmol, 1 eq., 1 M in THF) and *n*-butyllithium (1.44 ml, 3.60 mmol, 1 eq., 2.5 M in hexane) were added to give a dark red solution, which was allowed to warm up to –50 °C. It was lyered with *n*-pentane (1 ml) and stored at –80 °C. After two days red block-shaped crystals of compound **5** formed, suitable for single crystal X-ray analysis. The mother liquor was removed and the crystals were washed with cold *n*-pentane (3 x 1 ml) until the solvent remained colorless. Subsequently, the crystals were carefully dried in the vacuum, but due to the weak THF metal bond decomposition of the crystals at room temperature occurred. At lower temperatures a yield of 82% (937 mg, 2.95 mmol) could be estimated. The results of the NMR studies are in good agreement with the data in the literature.[8]

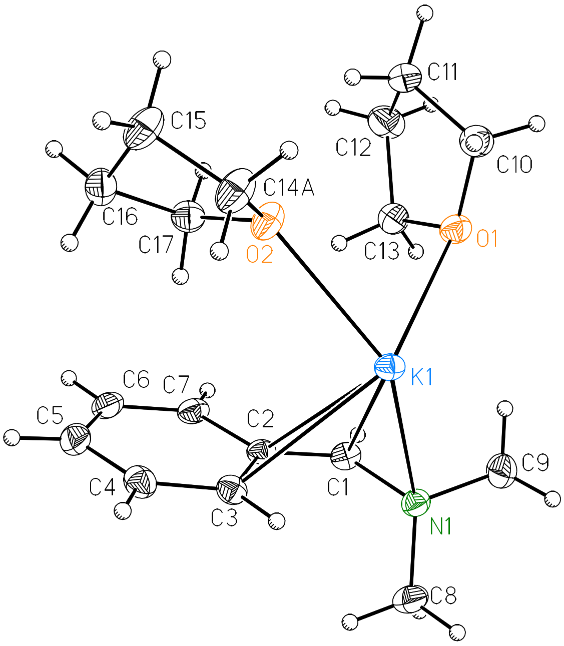

5

Figure S12 Ortep plot[5] of the asymmetric unit of **5** in the crystal with displacement ellipsoids drawn at the 50% probability level. Hydrogen atoms and disorders are omitted for clarity. Selected bond lengths [Å] and angles [°]: K1–O1 2.719(1), K1–O2 2.712(1), K1–N1 2.825(1), K1–C1 3.026(1), K1–C2 3.113(1), K1–C3 3.001(1), K1–C2‘

3.118(1), K1–C3‘ 3.097(1), K1–C4‘ 3.144(1), K1–C5‘ 3.163(1), K1–C6‘ 3.111(1), K1–C7‘ 3.084(1), C1–C2 1.380(1), C2–C3 1.449(1), C2–C7 1.446(1), C3–C4 1.386(1), C4–C5 1.398(1), C5–C6 1.409(1), C6–C7 1.373(1); O2–K1–N1 140.4(1), O2–K1–C1 112.4(1), N1–K1–C1 28.1(1), C1–C2–K1 73.5(1), C1–C2–C3 124.1(1), C1–C2–C7 121.7(1), C7–C2–C3 114.2(1), C4–C3–C2 121.8(1), C3–C4–C5 122.4(1), C4–C5–C6 117.0(1), C7–C6–C5 122.1(1).

Table S2 Crystal data and structure refinement of (*rac*)-**3**, (R_p)-**3** and **4**.

Compound	(*rac*)–**3**[a]	(R_p)–**3**	**4**[b]
Empirical formula	$C_{46}H_{74}Fe_2K_2Li_2N_2O_5$	$C_{46}H_{74}Fe_2K_2Li_2N_2O_5$	$C_{31}H_{57}K_3O_6$
Formula weight [g·mol^{-1}]	938.85	938.85	643.06
Temperature [K]	100.0	100.0	100.0
Wavelength [Å]	0.71073	0.71073	0.71073
Crystal System	monoclinic	monoclinic	triclinic
Space Group (Nr.)	*C*2	*C*2	$P\bar{1}$
a [Å]	13.1476(8)	13.1182(4)	9.0384(7)
b [Å]	17.5529(11)	17.5657(5)	11.2518(9)
c [Å]	12.2361(7)	12.2444(4)	19.6097(15)
α [°]	90	90	99.724(3)
β [°]	111.206(2)	111.2160(10)	90.068(3)
γ [°]	90	90	112.820(3)
Volume [Å^3]	2632.6(3)	2630.25(14)	1806.9(2)
Z	2	2	2
Density (calculated) ρ [g·cm^{-3}]	1.184	1.185	1.182
Absorption coefficient μ [mm^{-1}]	0.749	0.750	0.414
F(000)	1000.0	1000.0	696.0
Crystal size [mm^3]	0.336 × 0.3 × 0.182	0.15 × 0.138 × 0.059	0.262 × 0.161 × 0.143
Theta range for data collection θ [°]	4.538 to 61.082	4.058 to 56.708	4.162 to 57.996
Index ranges	–18 ≤ h ≤ 18, –25 ≤ k ≤ 25, –16 ≤ l ≤ 17	–17 ≤ h ≤ 17, –23 ≤ k ≤ 23, –16 ≤ l ≤ 16	–12 ≤ h ≤ 12, –15 ≤ k ≤ 15, –26 ≤ l ≤ 26
Reflections collected	53464	33026	200907
Independent reflections	8044 [R_{int} = 0.0377, R_{sigma} = 0.0231]	6564 [R_{int} = 0.0419, R_{sigma} = 0.0304]	9603 [R_{int} = 0.0517, R_{sigma} = 0.0189]
Structural refinement	Full–matrix least –squares on F^2		
Data / restraints / parameters	8044/31/332	6564/37/319	9603/12/415
Goodness–of–fit on F^2	1.037	1.038	1.084
Final *R* indices [$I > 2\sigma(I)$]	R_1 = 0.0417, wR_2 = 0.1043	R_1 = 0.0393, wR_2 = 0.0883	R_1 = 0.0572, wR_2 = 0.1665
R indices (all data)	R_1 = 0.0516, wR_2 = 0.1120	R_1 = 0.0509, wR_2 = 0.0946	R_1 = 0.0616, wR_2 = 0.1731
absolute structure parameter	–[a]	–0.004(6)	–[b]
Largest diff. Peak and hole [e·Å^{-3}]	0.45/–0.49	0.47/–0.51	1.27–0.64

[a]Twin-Law for the inversion twin (–1, 0, 0, 0, –1, 0, 0, 0, –1) BASF [0.19(3)]; [b] Twin-Law for the twin (1, 0, 0, –1, –1, 0, 0, 0, –1) BASF [0.06(1)]

Table S3 Crystal data and structure refinement of **5**.

Compound	**5**
Empirical formula	$C_{17}H_{28}KNO_2$
Formula weight [g·mol^{-1}]	317.50
Temperature [K]	100.0
Wavelength [Å]	0.71073
Crystal System	monoclinic
Space Group (Nr.)	$P2_1/c$
a [Å]	9.031(2)
b [Å]	18.109(4)
c [Å]	11.088(3)
α [°]	90
β [°]	98.266(5)
γ [°]	90
Volume [Å^3]	1794.5(7)
Z	4
Density (calculated) *ρ* [g·cm^{-3}]	1.175
Absorption coefficient *μ* [mm^{-1}]	0.300
F(000)	688.0
Crystal size [mm^3]	1.275 × 1.204 × 0.5
Theta range for data collection *θ* [°]	4.34 to 104.854
Index ranges	$-20 \le h \le 20$, $-40 \le k \le 40$, $-24 \le l \le 24$
Reflections collected	840529
Independent reflections	20798 [R_{int} = 0.0345, R_{sigma} = 0.0072]
Structural refinement	Full-matrix least -squares on F^2
Data / restraints / parameters	20798/0/304
Goodness-of-fit an F^2	1.022
Final *R* indices [$I > 2\sigma(I)$]	R_1 = 0.0269, wR_2 = 0.0838
R indices (all data)	R_1 = 0.0320, wR_2 = 0.0888
absolute structure parameter	–
Largest diff. Peak and hole [e·Å^{-3}]	0.69/–0.28

4. Quantum Chemical Calculations

Optimization, relaxed potential energy surface scans and additional harmonic vibrational frequency analyses were performed with the software package Gaussian 16 (Revision B.01)[9] on the M062X[10]/6-31+G(d)[11] or M062X/def2-tzvpp[12] level of theory using Densitiy-Functional Theory (DFT).[13] The GJF input-files were created with the program GaussView 6.0[14] The ground state structures were optimized without symmetry restrictions. Vibrational frequency analysis showed no imaginary frequency in the harmonical approximation for the ground states. The calculated standard orientations of the optimized structures can be found in the following Tables.

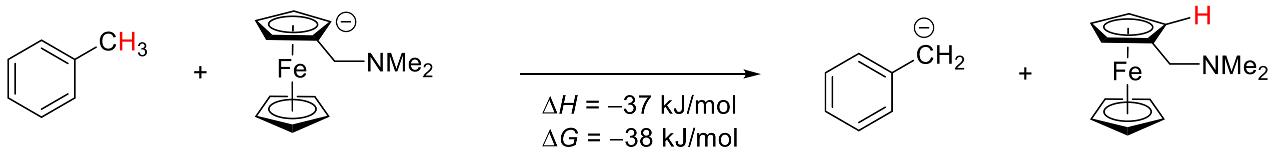

QM1 **QM6** **QM3** **QM5**

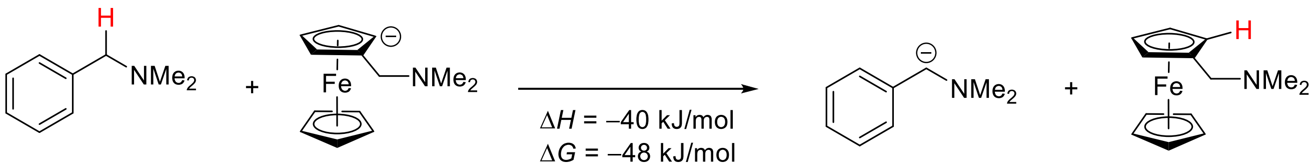

QM4 **QM6** **QM2** **QM5**

Scheme S1 Isodesmic reactions between Toluene or DBA with a ferrocenylanion derived from compound **3**.

Table S4 Total (SCF) and zero-point-corrected (ZPE) energies of the optimized structures of the isodesmic reaction between toluene/ferrocenylanion and the *N*,*N*-dimethylbenzylamine/ferrocenylanion.

Name	Calculation number	SCF [Hartree]	ZPE [Hartree]
**Toluene**	**QM1**	–271.44144992	–271.312248
**DBA-anion**	**QM2**	–1823.00565898	–1822.74623
**Benzylanion**	**QM3**	–270.82093089	–270.707497
**DBA**	**QM4**	–1823.63934498	–1823.365092
**Ferrocene**	**QM5**	–1823.407073	–1823.365092
**Ferrocenylanion**	**QM6**	–1823.005659	–1822.74623

Table S5 Coordinates of **QM1**.

Element	X	Y	Z
**C**	–0.194957	1.200673	–0.009237
**C**	1.198854	1.203754	0.002137
**C**	1.901213	–0.000190	0.008539
**C**	1.198398	–1.204009	0.002139
**C**	–0.195279	–1.200472	–0.009239
**C**	–0.911733	0.000277	–0.012085
**H**	–0.736315	2.144245	–0.017922
**H**	1.736486	2.147762	0.002243
**H**	2.987214	–0.000403	0.014434

**H**	1.735782	–2.148160	0.002243
**H**	–0.737017	–2.143849	–0.017933
**C**	–2.420177	0.000081	0.009989
**H**	–2.824684	–0.880172	–0.498307
**H**	–2.794741	–0.011625	1.040166
**H**	–2.824642	0.891512	–0.478381

Table S6 Coordinates of **QM2**.

Element	X	Y	Z
**C**	1.476985	–1.311095	–0.000008
**C**	0.275376	–0.50143	–0.000027
**C**	0.536552	0.922283	0.000871
**C**	1.822789	1.439	0.00061
**C**	2.96422	0.62381	–0.000154
**C**	2.744593	–0.769489	–0.000246
**H**	1.357017	–2.394719	0.000059
**H**	–0.301778	1.613244	0.001833
**H**	1.942543	2.52391	0.001171
**H**	3.966205	1.043247	–0.000348
**H**	3.600872	–1.446167	–0.000369
**C**	–0.984014	–1.085558	–0.000664
**H**	–1.067206	–2.1728	–0.001187
**C**	–2.569385	0.359284	–1.202396
**H**	–1.989505	1.293229	–1.334193
**H**	–2.378831	–0.275697	–2.071961
**H**	–3.637104	0.632635	–1.192494
**C**	–2.570593	0.357242	1.202323
**H**	–3.638592	0.629547	1.19242
**H**	–2.379697	–0.278769	2.071047
**H**	–1.991784	1.291646	1.335648
**N**	–2.237325	–0.380513	–0.000498

Table S7 Coordinates of **QM3**.

Element	X	Y	Z
**C**	0.243775	–1.210182	–0.000027
**C**	–1.137985	–1.197466	–0.000023
**C**	–1.877961	0.000000	–0.000002
**C**	–1.137985	1.197466	0.000008
**C**	0.243775	1.210182	0.000008
**C**	1.038331	–0.000000	0.000007
**H**	0.770652	–2.164708	–0.000051
**H**	–1.667847	–2.151932	–0.000045
**H**	–2.964312	0.000000	0.000010
**H**	–1.667847	2.151932	0.000015
**H**	0.770653	2.164708	0.000011
**C**	2.425265	0.000000	0.000026

**H**	2.987707	0.930185	0.000052
**H**	2.987707	–0.930184	0.000030

Table S8 Coordinates of **QM4**.

Element	X	Y	Z
**C**	0.910899	–1.201293	0.336045
**C**	0.250355	0.000003	0.611839
**C**	0.91091	1.201296	0.336044
**C**	2.193072	1.205264	–0.21051
**C**	2.836579	–0.000007	–0.487573
**C**	2.193063	–1.205271	–0.21051
**H**	0.41601	–2.144724	0.559307
**H**	0.416024	2.144728	0.559307
**H**	2.69165	2.148443	–0.415201
**H**	3.836878	–0.00001	–0.910663
**H**	2.691631	–2.148455	–0.415199
**C**	–1.166504	0.000014	1.157841
**H**	–1.314978	–0.883596	1.79028
**H**	–1.314969	0.88365	1.790248
**C**	–2.247314	–1.197954	–0.664354
**H**	–1.376978	–1.276481	–1.340273
**H**	–2.266462	–2.083767	–0.020419
**H**	–3.155494	–1.200385	–1.275581
**C**	–2.24731	1.197945	–0.664378
**H**	–3.155439	1.200314	–1.275679
**H**	–2.266558	2.083771	–0.020462
**H**	–1.376926	1.276501	–1.340233
**N**	–2.232412	0.000004	0.155415

Table S9 Coordinates of **QM5**.

Element	X	Y	Z
**Fe**	–1.143221	0.169331	–0.01921
**N**	3.278338	–0.351443	–0.312397
**C**	0.382325	1.176804	1.040963
**H**	0.564327	1.022376	2.097758
**C**	–0.504104	2.129549	0.470479
**H**	–1.121206	2.832063	1.014863
**C**	–0.475479	1.96006	–0.940805
**H**	–1.068153	2.511452	–1.65888
**C**	–2.089889	–1.477719	–0.956619
**H**	–1.75688	–1.88815	–1.900831
**C**	–1.626522	–1.861842	0.330968
**H**	–0.880024	–2.617394	0.539826
**C**	–2.279016	–1.045266	1.29445
**H**	–2.115133	–1.069695	2.363659
**C**	–3.145137	–0.156564	0.602304

H	–3.756039	0.614762	1.0523
C	–3.027906	–0.423115	–0.788889
H	–3.534652	0.10895	–1.583066
C	1.936917	–0.71727	0.132595
H	1.946893	–1.052205	1.191333
H	1.612413	–1.572256	–0.472969
C	3.813615	0.757681	0.458633
H	4.814521	1.003622	0.092326
H	3.886289	0.516997	1.538211
H	3.176049	1.637481	0.342538
C	4.161251	–1.500954	–0.235505
H	3.764268	–2.315828	–0.848834
H	4.280408	–1.872143	0.801577
H	5.149843	–1.232245	–0.618989
C	0.955041	0.409234	–0.012267
C	0.425365	0.902951	–1.237199
H	0.653696	0.508411	–2.219218

Table S10 Coordinates of **QM6**.

Element	X	Y	Z
Fe	0.954786	0.131517	–0.021428
C	0.152463	1.756439	–1.065316
H	–0.02841	1.724905	–2.137192
C	1.362999	2.13765	–0.430249
H	2.277875	2.443859	–0.930143
C	1.15062	1.964607	0.98016
H	1.933933	2.13297	1.717169
C	1.093147	–1.763769	0.961953
H	0.649654	–1.910303	1.938111
C	0.435373	–1.927242	–0.286324
H	–0.601617	–2.201231	–0.429032
C	1.335118	–1.528956	–1.311974
H	1.113392	–1.492074	–2.370977
C	2.551188	–1.118192	–0.696557
H	3.418554	–0.715665	–1.204538
C	2.399233	–1.262118	0.709545
H	3.126079	–0.979036	1.4601
C	–0.760802	1.361245	–0.025668
C	–0.171684	1.492174	1.284094
C	–2.186905	0.965637	–0.328765
H	–2.865961	1.583536	0.297454
H	–2.407972	1.208186	–1.377739
C	–3.860478	–0.711988	–0.665738
H	–4.106412	–1.773342	–0.539112
H	–4.643039	–0.117336	–0.145746
H	–3.904825	–0.472024	–1.734342
C	–2.454539	–0.843978	1.246492

**H**	–1.494034	–0.525781	1.660961
**H**	–3.249999	–0.358836	1.850162
**H**	–2.570367	–1.933536	1.329134
**N**	–2.535098	–0.451824	–0.151007

Table S11 Total (SCF) and zero-point-corrected (ZPE) energies of the optimized structures of the THF detachment vs. displacement studies of compound **3**.

Name	Calculation number	SCF [Hartree]	ZPE [Hartree]
**initial structure**	**QM7**	–2953.962707	–2953.07806
**DME**	**QM8**	–154.8249122	–154.744548
**DME side–detachment**	**QM9**	–2799.118902	–2798.3155
**DME bridge–detachment**	**QM10**	–2799.112565	–2798.309618
**DME displacement**	**QM11**	–2953.95417	–2953.069394
**DME side-detachment (3.8 Å)**	**QM12**	–2953.956292	–2953.072022
**DME side-detachment (4.8 Å)**	**QM13**	–2953.952927	–2953.068229

Table S12 Coordinates of **QM7**.

Element	X	Y	Z
**K**	–0.463764	1.69589	0.308816
**O**	–0.531458	1.306866	–2.212942
**O**	–2.015206	3.564036	1.497056
**N**	1.162458	3.893542	0.468234
**C**	2.352773	3.409654	–0.236976
**H**	3.147768	4.188824	–0.20353
**H**	2.065497	3.277602	–1.291149
**C**	1.3974	4.170229	1.869684
**H**	0.45575	4.500015	2.334522
**H**	2.157061	4.968897	2.02109
**H**	1.753264	3.267651	2.386211
**C**	0.605562	5.057576	–0.191089
**H**	0.429753	4.837613	–1.253511
**H**	1.282884	5.936943	–0.131954
**H**	–0.354629	5.323141	0.276182
**C**	–1.098941	2.133712	–3.168689
**C**	0	2.888842	–3.931366
**H**	0.599835	3.481089	–3.222127
**H**	–0.403647	3.567142	–4.699146
**H**	0.671973	2.168315	–4.424712
**C**	–1.918964	1.295084	–4.164937
**H**	–1.261864	0.555613	–4.650741
**H**	–2.392404	1.904705	–4.950013
**H**	–2.718518	0.755832	–3.628159
**C**	–2.031736	3.153174	–2.491507
**H**	–2.832478	2.619868	–1.954369
**H**	–2.498166	3.84684	–3.20875

**H**	–1.457568	3.747777	–1.761495
**Li**	1.132474	0.479476	–2.011631
**K**	0.463764	–1.69589	0.308816
**O**	0.531458	–1.306866	–2.212942
**O**	2.015206	–3.564036	1.497056
**N**	–1.162458	–3.893542	0.468234
**C**	–2.352773	–3.409654	–0.236976
**H**	–3.147768	–4.188824	–0.20353
**H**	–2.065497	–3.277602	–1.291149
**C**	–1.3974	–4.170229	1.869684
**H**	–0.45575	–4.500015	2.334522
**H**	–2.157061	–4.968897	2.02109
**H**	–1.753264	–3.267651	2.386211
**C**	–0.605562	–5.057576	–0.191089
**H**	–0.429753	–4.837613	–1.253511
**H**	–1.282884	–5.936943	–0.131954
**H**	0.354629	–5.323141	0.276182
**C**	1.098941	–2.133712	–3.168689
**C**	0	–2.888842	–3.931366
**H**	–0.599835	–3.481089	–3.222127
**H**	0.403647	–3.567142	–4.699146
**H**	–0.671973	–2.168315	–4.424712
**C**	1.918964	–1.295084	–4.164937
**H**	1.261864	–0.555613	–4.650741
**H**	2.392404	–1.904705	–4.950013
**H**	2.718518	–0.755832	–3.628159
**C**	2.031736	–3.153174	–2.491507
**H**	2.832478	–2.619868	–1.954369
**H**	2.498166	–3.84684	–3.20875
**H**	1.457568	–3.747777	–1.761495
**Li**	–1.132474	–0.479476	–2.011631
**C**	3.207293	–4.105269	0.987126
**H**	2.992149	–4.512176	–0.008289
**H**	3.98983	–3.331028	0.894248
**H**	3.583551	–4.914699	1.637746
**C**	2.164831	–3.022657	2.782272
**H**	2.505162	–3.787988	3.502823
**H**	2.882478	–2.181994	2.782525
**H**	1.180947	–2.652527	3.10673
**C**	–3.207293	4.105269	0.987126
**H**	–2.992149	4.512176	–0.008289
**H**	–3.98983	3.331028	0.894248
**H**	–3.583551	4.914699	1.637746
**C**	–2.164831	3.022657	2.782272
**H**	–2.505162	3.787988	3.502823
**H**	–2.882478	2.181994	2.782525
**H**	–1.180947	2.652527	3.10673
**O**	0	0	2.437379

**C**	1.088161	0.459373	3.208421
**H**	1.897461	0.745391	2.520351
**H**	0.793111	1.330352	3.822384
**H**	1.451645	–0.331654	3.887886
**C**	–1.088161	–0.459373	3.208421
**H**	–1.897461	–0.745391	2.520351
**H**	–0.793111	–1.330352	3.822384
**H**	–1.451645	0.331654	3.887886
**C**	–2.912032	–2.090049	0.270524
**C**	–2.441107	–0.8564	–0.262462
**C**	–3.940806	–2.16426	1.224066
**C**	–3.155493	0.267249	0.219379
**C**	–4.576479	–1.014914	1.691134
**H**	–4.267629	–3.143071	1.590751
**C**	–4.192026	0.214039	1.160387
**H**	–2.934283	1.264057	–0.192191
**H**	–5.381857	–1.083568	2.424842
**H**	–4.717323	1.125124	1.463354
**C**	2.912032	2.090049	0.270524
**C**	2.441107	0.8564	–0.262462
**C**	3.940806	2.16426	1.224066
**C**	3.155493	–0.267249	0.219379
**C**	4.576479	1.014914	1.691134
**H**	4.267629	3.143071	1.590751
**C**	4.192026	–0.214039	1.160387
**H**	2.934283	–1.264057	–0.192191
**H**	5.381857	1.083568	2.424842
**H**	4.717323	–1.125124	1.463354

Table S13 Coordinates of **QM8**.

Element	X	Y	Z
**O**	0	0.58211	0
**C**	1.160771	-0.189937	0
**H**	2.022673	0.489004	-0.000003
**H**	1.221824	-0.83891	0.894851
**H**	1.221822	-0.838915	-0.894848
**C**	-1.160771	-0.189937	0
**H**	-2.022673	0.489004	0.000003
**H**	-1.221824	-0.83891	-0.894851
**H**	-1.221822	-0.838915	0.894848

Table S14 Coordinates of **QM9**.

Element	X	Y	Z
**K**	–1.324242	0.643509	0.143421
**O**	–0.639946	–0.851005	2.174119
**O**	–2.779788	2.90859	0.299534

N	–3.856793	–0.197735	–0.514759
C	–3.693009	–1.631363	–0.7683
H	–4.636865	–2.043202	–1.192018
H	–3.53457	–2.113767	0.207951
C	–4.120827	0.561357	–1.720312
H	–4.211688	1.62687	–1.462142
H	–5.059991	0.240731	–2.222752
H	–3.299438	0.437185	–2.440879
C	–4.895692	0.04143	0.467218
H	–4.691212	–0.534973	1.380484
H	–5.9002	–0.253907	0.094183
H	–4.920834	1.110842	0.726139
C	–1.421353	–0.740657	3.312412
C	–2.368696	–1.945514	3.415708
H	–2.997767	–1.995007	2.512319
H	–3.025709	–1.895762	4.297994
H	–1.783166	–2.876609	3.471745
C	–0.522763	–0.689754	4.558246
H	0.088986	–1.604263	4.606681
H	–1.09667	–0.60536	5.494232
H	0.155313	0.176772	4.489214
C	–2.26276	0.548811	3.257374
H	–1.594941	1.421919	3.17636
H	–2.893169	0.680338	4.150728
H	–2.927762	0.529111	2.37717
Li	–0.347443	–2.149429	0.873739
K	1.67568	–0.565599	–1.211135
O	1.576948	–2.005462	0.839946
N	4.17761	0.595666	–1.125207
C	4.014086	0.974236	0.288888
H	4.984914	1.35683	0.675777
H	3.783559	0.049401	0.840611
C	4.596422	1.705495	–1.959779
H	4.70083	1.367533	–3.001413
H	5.573406	2.12476	–1.63521
H	3.853105	2.512076	–1.926922
C	5.120648	–0.504361	–1.231397
H	4.770903	–1.35813	–0.631873
H	6.131192	–0.225427	–0.865595
H	5.214727	–0.82696	–2.278891
C	2.190963	–3.12423	1.392083
C	3.234969	–2.690143	2.432821
H	3.992589	–2.049064	1.954741
H	3.749014	–3.544185	2.900279
H	2.748387	–2.107583	3.23355
C	1.141317	–4.015684	2.083207
H	0.626697	–3.441162	2.8704
H	1.581973	–4.913651	2.541913

**H**	0.391969	–4.357086	1.346535
**C**	2.884758	–3.941731	0.292087
**H**	2.143055	–4.266621	–0.453798
**H**	3.393379	–4.834821	0.68725
**H**	3.636043	–3.317445	–0.218246
**Li**	1.140681	–0.455643	1.835976
**C**	–3.040631	3.667016	1.452308
**H**	–3.482205	2.998789	2.201809
**H**	–2.111258	4.101165	1.863383
**H**	–3.748213	4.486698	1.234337
**C**	–2.233189	3.670036	–0.746485
**H**	–2.915216	4.487099	–1.042973
**H**	–1.256143	4.099961	–0.459934
**H**	–2.087208	3.000608	–1.606593
**O**	–0.061878	1.334268	–2.189105
**C**	–0.582758	0.80289	–3.388864
**H**	–1.1074	–0.133296	–3.149602
**H**	–1.283847	1.514162	–3.861327
**H**	0.22844	0.584467	–4.107251
**C**	0.689902	2.51364	–2.375641
**H**	1.05238	2.846937	–1.392564
**H**	1.552697	2.323345	–3.040135
**H**	0.070234	3.305499	–2.833126
**C**	2.911882	1.980983	0.575443
**C**	1.629194	1.531949	0.997004
**C**	3.243791	3.342295	0.468609
**C**	0.74357	2.588124	1.319534
**C**	2.312196	4.335646	0.762586
**H**	4.257411	3.632532	0.173677
**C**	1.050141	3.949548	1.209177
**H**	–0.252736	2.357526	1.726348
**H**	2.580246	5.390587	0.680576
**H**	0.31746	4.710365	1.495175
**C**	–2.528123	–2.002474	–1.672579
**C**	–1.219704	–2.156835	–1.133995
**C**	–2.825567	–2.251133	–3.022249
**C**	–0.284259	–2.657653	–2.070716
**C**	–1.842883	–2.689193	–3.910418
**H**	–3.851446	–2.118563	–3.381989
**C**	–0.560358	–2.920223	–3.419063
**H**	0.728864	–2.909247	–1.721133
**H**	–2.087038	–2.88333	–4.956615
**H**	0.215876	–3.321454	–4.076772

Table S15 Coordinates of **QM10**.

Element	X	Y	Z
**K**	1.572995	–0.841491	–0.240088
**O**	1.205917	–0.739554	2.29448
**O**	1.885572	–2.530609	–2.29109
**N**	3.201083	0.800071	–1.732667
**C**	3.396356	1.76217	–0.643271
**H**	4.324595	2.348395	–0.824709
**H**	3.565339	1.184252	0.282315
**C**	3.012148	1.444395	–3.01671
**H**	2.79379	0.685927	–3.783083
**H**	3.914	2.010856	–3.33585
**H**	2.173025	2.148955	–2.964189
**C**	4.299096	–0.145497	–1.796587
**H**	4.420579	–0.659794	–0.828854
**H**	5.267136	0.346198	–2.030618
**H**	4.092469	–0.904403	–2.564728
**C**	2.1479	–1.21893	3.19121
**C**	2.634486	–0.07493	4.095707
**H**	3.072191	0.726577	3.477367
**H**	3.392572	–0.398936	4.825452
**H**	1.778674	0.342528	4.650224
**C**	1.533676	–2.325725	4.062342
**H**	0.673437	–1.919199	4.617719
**H**	2.249648	–2.738101	4.790075
**H**	1.172575	–3.148115	3.424788
**C**	3.356506	–1.794142	2.42806
**H**	3.030483	–2.614491	1.765737
**H**	4.131959	–2.192664	3.099898
**H**	3.819275	–1.002649	1.813901
**Li**	0.628697	1.025029	2.075522
**K**	–1.572995	0.841491	–0.240088
**O**	–1.205917	0.739554	2.29448
**O**	–1.885572	2.530609	–2.29109
**N**	–3.201083	–0.800071	–1.732667
**C**	–3.396356	–1.76217	–0.643271
**H**	–4.324595	–2.348395	–0.824709
**H**	–3.565339	–1.184252	0.282315
**C**	–3.012148	–1.444395	–3.01671
**H**	–2.79379	–0.685927	–3.783083
**H**	–3.914	–2.010856	–3.33585
**H**	–2.173025	–2.148955	–2.964189
**C**	–4.299096	0.145497	–1.796587
**H**	–4.420579	0.659794	–0.828854
**H**	–5.267136	–0.346198	–2.030618
**H**	–4.092469	0.904403	–2.564728
**C**	–2.1479	1.21893	3.19121
**C**	–2.634486	0.07493	4.095707

**H**	–3.072191	–0.726577	3.477367
**H**	–3.392572	0.398936	4.825452
**H**	–1.778674	–0.342528	4.650224
**C**	–1.533676	2.325725	4.062342
**H**	–0.673437	1.919199	4.617719
**H**	–2.249648	2.738101	4.790075
**H**	–1.172575	3.148115	3.424788
**C**	–3.356506	1.794142	2.42806
**H**	–3.030483	2.614491	1.765737
**H**	–4.131959	2.192664	3.099898
**H**	–3.819275	1.002649	1.813901
**Li**	–0.628697	–1.025029	2.075522
**C**	–2.499214	3.793026	–2.310476
**H**	–3.449384	3.714532	–1.766088
**H**	–1.860852	4.548605	–1.821761
**H**	–2.706913	4.11653	–3.346279
**C**	–0.628697	2.529621	–2.923099
**H**	–0.722904	2.810304	–3.9878
**H**	0.071857	3.219716	–2.419187
**H**	–0.223341	1.508728	–2.867849
**C**	2.499214	–3.793026	–2.310476
**H**	3.449384	–3.714532	–1.766088
**H**	1.860852	–4.548605	–1.821761
**H**	2.706913	–4.11653	–3.346279
**C**	0.628697	–2.529621	–2.923099
**H**	0.722904	–2.810304	–3.9878
**H**	–0.071857	–3.219716	–2.419187
**H**	0.223341	–1.508728	–2.867849
**C**	–2.225756	–2.702733	–0.414471
**C**	–1.077018	–2.260205	0.29698
**C**	–2.359604	–4.011097	–0.901862
**C**	–0.108663	–3.273974	0.488254
**C**	–1.364769	–4.965934	–0.686128
**H**	–3.26731	–4.296197	–1.44389
**C**	–0.232132	–4.593529	0.031641
**H**	0.798185	–3.04198	1.067583
**H**	–1.487097	–5.986493	–1.054052
**H**	0.547109	–5.329182	0.250996

Table S16 Coordinates of **QM11**.

Element	X	Y	Z
**K**	–1.671402	0.316911	–0.468545
**O**	–1.473575	–0.097423	2.040582
**O**	–4.075554	1.439187	–1.12181
**N**	–3.501495	–1.728534	–0.739082
**C**	–2.732025	–2.878956	–0.265742
**H**	–3.331329	–3.808926	–0.398201

H	–2.587007	–2.736005	0.815934
C	–3.806255	–1.770197	–2.153593
H	–4.381661	–0.871247	–2.419395
H	–4.406492	–2.665034	–2.431917
H	–2.883583	–1.784059	–2.750563
C	–4.715741	–1.579727	0.0353
H	–4.469778	–1.536644	1.105305
H	–5.417457	–2.428081	–0.121446
H	–5.223785	–0.647387	–0.250067
C	–2.208017	0.025296	3.213528
C	–2.554571	–1.365813	3.767782
H	–3.13537	–1.92762	3.019434
H	–3.140466	–1.32021	4.698934
H	–1.628193	–1.927293	3.97604
C	–1.390757	0.791825	4.273194
H	–0.451502	0.252442	4.480595
H	–1.932692	0.913419	5.223077
H	–1.147915	1.803847	3.901204
C	–3.507405	0.798025	2.942131
H	–3.268776	1.807052	2.570105
H	–4.135337	0.899114	3.841421
H	–4.085243	0.273966	2.165947
Li	–0.276802	–1.514137	1.681504
K	1.911868	–0.159521	–0.05327
O	1.270572	–0.668203	2.366621
O	4.097546	–1.342548	–1.15076
N	3.486147	2.065966	–0.742197
C	2.868186	2.823462	0.35948
H	3.483413	3.727968	0.563418
H	2.924401	2.193583	1.26229
C	3.591879	2.85624	–1.95453
H	4.036239	2.250084	–2.758683
H	4.233212	3.752346	–1.808839
H	2.601677	3.199483	–2.278839
C	4.804731	1.606767	–0.335647
H	4.727815	0.962066	0.552626
H	5.476627	2.455309	–0.08745
H	5.267	1.014943	–1.137625
C	2.254698	–1.205866	3.179866
C	2.903055	–0.098699	4.023681
H	3.331408	0.670118	3.360386
H	3.702626	–0.47911	4.678259
H	2.139351	0.384863	4.652515
C	1.642738	–2.262789	4.113488
H	0.85619	–1.798576	4.727982
H	2.386947	–2.717766	4.785407
H	1.183629	–3.067434	3.515246
C	3.338616	–1.879753	2.31659

H	2.867107	–2.626505	1.655773
H	4.108122	–2.38503	2.920933
H	3.851855	–1.129304	1.689441
Li	0.184043	0.824652	2.205253
C	4.714151	–2.590563	–0.956831
H	4.898687	–2.71047	0.117161
H	4.063335	–3.413814	–1.302172
H	5.674525	–2.642762	–1.499299
C	3.847892	–1.059566	–2.501761
H	4.782655	–1.060125	–3.091211
H	3.14803	–1.793913	–2.941385
H	3.403032	–0.053974	–2.555206
C	–4.867464	2.246948	–0.291544
H	–5.134504	1.6607	0.595851
H	–4.316373	3.149085	0.03138
H	–5.790394	2.564237	–0.80966
C	–3.689937	2.09178	–2.305466
H	–4.570114	2.364409	–2.915316
H	–3.115561	3.01061	–2.082857
H	–3.051128	1.406345	–2.879976
O	–0.68993	0.553508	–2.912386
C	–0.130784	–0.535719	–3.605415
H	–0.683197	–1.444487	–3.328957
H	–0.183119	–0.38033	–4.697609
H	0.926278	–0.685011	–3.319349
C	0.061515	1.736694	–3.024944
H	–0.399713	2.507721	–2.388642
H	1.098859	1.579836	–2.672005
H	0.095502	2.092298	–4.070368
C	1.416872	3.224864	0.158211
C	0.375188	2.408886	0.676271
C	1.173499	4.453408	–0.478416
C	–0.912743	2.981404	0.534163
C	–0.12285	4.941721	–0.630345
H	2.01519	5.055165	–0.836904
C	–1.176972	4.203273	–0.096046
H	–1.776773	2.458958	0.973174
H	–0.30055	5.901169	–1.119829
H	–2.198021	4.593096	–0.147555
C	–1.371925	–3.06394	–0.917279
C	–0.206829	–2.469793	–0.350857
C	–1.330872	–3.901251	–2.044302
C	0.984825	–2.866191	–1.006842
C	–0.127302	–4.209791	–2.674871
H	–2.260742	–4.338701	–2.422223
C	1.047653	–3.700184	–2.130401
H	1.962238	–2.557133	–0.609471
H	–0.106713	–4.865454	–3.54739

**H**	2.013637	–3.972752	–2.567363

Table S17 Coordinates of **QM12**.

Element	X	Y	Z
**K**	–1.789558	0.08882	–0.398822
**O**	–1.613989	0.208592	2.209731
**O**	–3.761003	1.14958	–1.897131
**N**	–3.391654	–2.083964	–0.895625
**C**	–2.731972	–3.197489	–0.209924
**H**	–3.23772	–4.151813	–0.481228
**H**	–2.881096	–3.043785	0.869391
**C**	–3.311031	–2.18389	–2.338852
**H**	–3.80653	–1.310694	–2.788597
**H**	–3.802853	–3.103734	–2.725244
**H**	–2.260982	–2.205424	–2.665009
**C**	–4.773152	–1.958511	–0.475518
**H**	–4.828447	–1.880488	0.619528
**H**	–5.386835	–2.831146	–0.787653
**H**	–5.211775	–1.049344	–0.914137
**C**	–2.80198	0.40401	2.894775
**C**	–3.292872	–0.927833	3.482719
**H**	–3.422367	–1.660544	2.669996
**H**	–4.248369	–0.828642	4.021019
**H**	–2.541953	–1.327771	4.182084
**C**	–2.587821	1.414734	4.032305
**H**	–1.815107	1.034447	4.71871
**H**	–3.504904	1.604379	4.611633
**H**	–2.237769	2.372842	3.614351
**C**	–3.883035	0.951277	1.943161
**H**	–3.540926	1.901963	1.503187
**H**	–4.841495	1.133968	2.453774
**H**	–4.069852	0.230477	1.128764
**Li**	–0.423609	–1.210195	2.009561
**K**	1.669563	–0.006213	–0.211704
**O**	1.190862	–0.182456	2.245362
**O**	4.886248	–1.483819	–1.300486
**N**	3.40883	2.08804	–0.473902
**C**	2.624569	2.970039	0.402983
**H**	3.206827	3.895297	0.612514
**H**	2.499331	2.438513	1.35945
**C**	3.778367	2.713033	–1.727634
**H**	4.333841	1.993383	–2.34739
**H**	4.422797	3.606221	–1.573978
**H**	2.883185	3.032089	–2.276686
**C**	4.589068	1.596418	0.215692
**H**	4.291159	1.104166	1.15461
**H**	5.293934	2.414629	0.475689

**H**	5.112474	0.861823	–0.414191
**C**	1.779911	–0.436109	3.478414
**C**	1.942574	0.875706	4.263014
**H**	2.560367	1.579705	3.683271
**H**	2.415005	0.727805	5.246476
**H**	0.955312	1.339373	4.430228
**C**	0.898868	–1.400107	4.295966
**H**	–0.095882	–0.950265	4.450965
**H**	1.325522	–1.634208	5.282891
**H**	0.778951	–2.353315	3.75082
**C**	3.161317	–1.076766	3.278839
**H**	3.055296	–2.010868	2.704699
**H**	3.663129	–1.307379	4.231513
**H**	3.805477	–0.387798	2.709133
**Li**	–0.065734	1.22148	2.024247
**C**	5.08432	–2.165531	–0.092
**H**	4.790052	–1.497466	0.727796
**H**	4.469299	–3.08265	–0.037459
**H**	6.144647	–2.448265	0.041228
**C**	5.229278	–2.248384	–2.420101
**H**	6.29376	–2.545372	–2.394006
**H**	4.614696	–3.164673	–2.488555
**H**	5.055241	–1.637517	–3.314726
**C**	–4.706659	2.101137	–1.482337
**H**	–5.191861	1.720447	–0.575126
**H**	–4.224422	3.068134	–1.25134
**H**	–5.471851	2.264124	–2.26216
**C**	–3.091664	1.519163	–3.075926
**H**	–3.801405	1.645071	–3.913273
**H**	–2.526541	2.458504	–2.936395
**H**	–2.385329	0.714485	–3.325451
**O**	0.041292	0.115424	–2.421891
**C**	0.385699	–1.075022	–3.097356
**H**	0.071891	–1.925128	–2.475087
**H**	–0.114357	–1.126923	–4.080894
**H**	1.478631	–1.13439	–3.256387
**C**	0.448662	1.284028	–3.100237
**H**	0.158756	2.152398	–2.491193
**H**	1.545208	1.28872	–3.239622
**H**	–0.029244	1.345776	–4.094404
**C**	1.243389	3.331052	–0.120101
**C**	0.107246	2.570337	0.276411
**C**	1.150013	4.457093	–0.954535
**C**	–1.112336	3.078284	–0.231624
**C**	–0.078846	4.883955	–1.454654
**H**	2.053034	5.025046	–1.201177
**C**	–1.225832	4.191399	–1.073737
**H**	–2.06279	2.610646	0.067557

H	–0.141396	5.762379	–2.099666
H	–2.207766	4.536229	–1.412014
C	–1.238591	–3.325569	–0.466226
C	–0.309689	–2.558843	0.292046
C	–0.837351	–4.27519	–1.419621
C	1.03967	–2.908708	0.045206
C	0.512638	–4.53355	–1.658321
H	–1.596231	–4.837811	–1.973637
C	1.461441	–3.857116	–0.895659
H	1.827391	–2.438543	0.655027
H	0.813035	–5.276279	–2.399932
H	2.525008	–4.079598	–1.022105

Table S18 Coordinates of **QM13**.

Element	X	Y	Z
K	–1.716225	–0.403581	–0.591081
O	–2.031513	1.931239	0.520583
O	–3.066059	–1.360813	–2.718754
N	–3.627576	–1.970029	0.617899
C	–3.319445	–1.86424	2.046553
H	–3.946445	–2.58807	2.614852
H	–3.617107	–0.853427	2.364007
C	–3.311511	–3.272313	0.066348
H	–3.544681	–3.274302	–1.008799
H	–3.890041	–4.087934	0.5534
H	–2.243397	–3.497269	0.199866
C	–5.016198	–1.64056	0.364211
H	–5.252066	–0.652852	0.784663
H	–5.710696	–2.380029	0.818309
H	–5.200189	–1.611899	–0.720495
C	–3.249715	2.55993	0.322627
C	–4.096362	2.472684	1.601449
H	–4.245208	1.414306	1.870444
H	–5.083481	2.948335	1.492308
H	–3.56503	2.962649	2.432567
C	–3.021936	4.036221	–0.039749
H	–2.469586	4.532849	0.773431
H	–3.962358	4.584398	–0.205892
H	–2.417205	4.101236	–0.95934
C	–4.016929	1.882336	–0.82882
H	–3.420019	1.939192	–1.753765
H	–4.993195	2.353825	–1.02182
H	–4.197044	0.820853	–0.588643
Li	–1.141202	1.181237	1.974957
K	1.544306	0.033379	0.516628
O	0.553403	2.031157	1.669187
O	5.861269	–1.825326	0.695101

**N**	3.77191	0.921454	–0.848061
**C**	2.974505	2.032539	–1.395393
**H**	3.65628	2.744899	–1.911426
**H**	2.538494	2.566975	–0.536159
**C**	4.505282	0.192596	–1.865783
**H**	5.105302	–0.591741	–1.381282
**H**	5.198	0.852873	–2.431887
**H**	3.815464	–0.269757	–2.583892
**C**	4.690249	1.426863	0.161392
**H**	4.127294	1.885008	0.989687
**H**	5.370092	2.202166	–0.251005
**H**	5.308234	0.605195	0.551706
**C**	0.904569	2.970011	2.631752
**C**	1.471705	4.227955	1.956043
**H**	2.361849	3.96323	1.36376
**H**	1.756763	5.006272	2.68068
**H**	0.72104	4.657899	1.271606
**C**	–0.33288	3.36415	3.459965
**H**	–1.111321	3.770674	2.794841
**H**	–0.105996	4.115155	4.231867
**H**	–0.741724	2.476448	3.97499
**C**	1.965198	2.382714	3.575565
**H**	1.55912	1.495971	4.08625
**H**	2.295823	3.10266	4.340318
**H**	2.847968	2.07051	2.993295
**Li**	–0.305847	2.416246	0.028857
**C**	4.525178	–2.039914	1.04013
**H**	4.215966	–1.234164	1.72094
**H**	3.871321	–2.027504	0.143428
**H**	4.384736	–3.00783	1.556052
**C**	6.380518	–2.835711	–0.120455
**H**	6.337677	–3.820256	0.380089
**H**	5.827507	–2.909072	–1.076775
**H**	7.427727	–2.592182	–0.33605
**C**	–3.836855	–0.602535	–3.614813
**H**	–4.581893	–0.047675	–3.031507
**H**	–3.209576	0.116794	–4.171608
**H**	–4.356074	–1.254836	–4.339342
**C**	–2.091338	–2.144125	–3.358292
**H**	–2.552787	–2.847759	–4.07423
**H**	–1.358112	–1.512757	–3.892637
**H**	–1.565652	–2.720656	–2.583503
**O**	0.487536	–1.982637	–1.022716
**C**	0.593084	–3.176367	–0.277464
**H**	0.026267	–3.04889	0.655635
**H**	0.188913	–4.032032	–0.848038
**H**	1.647973	–3.392048	–0.027299
**C**	1.237176	–1.997667	–2.217728

**H**	1.084186	–1.037332	–2.730625
**H**	2.31362	–2.127258	–1.999027
**H**	0.914975	–2.827229	–2.872357
**C**	1.840772	1.639138	–2.33031
**C**	0.507693	1.547629	–1.839611
**C**	2.165204	1.444051	–3.683653
**C**	–0.445183	1.262395	–2.846573
**C**	1.184371	1.12708	–4.621218
**H**	3.200819	1.565989	–4.016643
**C**	–0.140397	1.049239	–4.196241
**H**	–1.515183	1.238303	–2.588343
**H**	1.446242	0.980936	–5.670802
**H**	–0.934376	0.850317	–4.922516
**C**	–1.858033	–2.064386	2.416247
**C**	–0.943999	–0.975208	2.355204
**C**	–1.49028	–3.33675	2.883981
**C**	0.331729	–1.285917	2.885094
**C**	–0.196929	–3.5944	3.338785
**H**	–2.236524	–4.137831	2.911769
**C**	0.718386	–2.544739	3.363064
**H**	1.075103	–0.479654	2.981873
**H**	0.075516	–4.586463	3.70406
**H**	1.721092	–2.698944	3.772249

Table S19 Total free GIBBS energies of the optimized structures of the THF detachment vs. displacement studies of compound **3**.

**Name**	**Calculation number**	**$\Delta G_{298\,K}$ [kJ/mol]**	**$\Delta G_{248\,K}$ [kJ/mol]**	**ΔG_{218} K [kJ/mol]**
**initial structure**	**QM7**	–7753704.165	–7753637.433	–7753597.395
**DME**	**QM8**	–406362.0594	–406348.595	–406340.516
**DME side–detachment**	**QM9**	–7347347.436	–7347285.340	–7347248.083
**DME bridge–detachment**	**QM10**	–7347340.673	–7347277.121	–7347238.990
**DME displacement**	**QM11**	–7753693.412	–7753624.667	–7753583.420
**DME side-detachment (3.8 Å)**	**QM12**	–7753698.605	–7753630.146	–7753589.071
**DME side-detachment (4.8 Å)**	**QM13**	–7753693.964	–7753624.613	–7753583.003

5. References

[1] T. Kottke, D. Stalke, *J. Appl. Cryst.* **1993**, *26*, 615–619.
[2] O. V. Dolomanov, L. J. Bourhis, R. J. Gildea, J. A. K. Howard, H. Puschmann, *J. Appl. Cryst.* **2009**, *42*, 339–341.
[3] a) G. M. Sheldrick, *Acta Cryst.* **2015**, *A71*, 3–8; b) G. M. Sheldrick, *Acta Cryst.* **2008**, *A64*, 112–122.
[4] G. M. Sheldrick, *Acta Cryst.* **2015**, *C71*, 3–8.
[5] L. J. Farrugia, *J. Appl. Cryst.* **1997**, *30*, 565.
[6] C. Unkelbach, D. F. O'Shea, C. Strohmann, *Angew. Chem. Int. Ed.* **2014**, *53*, 553–556; *Angew*. *Chem*. **2014**, *126*, 563–567.
[7] L. Lochmann, J. Trekoval, *J. Organomet. Chem.* **1987**, *326*, 1–7.
[8] F. T. Oakes, J. F. Sebastian, *J. Organomet. Chem.* **1978**, *159*, 363–371.
[9] M. J. Frisch, G. W. Trucks, H. B. Schlegel, G. E. Scuseria, M. A. Robb, J. R. Cheeseman, G. Scalmani, V. Barone, G. A. Petersson, H. Nakatsuji, X. Li, M. Caricato, A. V. Marenich, J. Bloino, B. G. Janesko, R. Gomperts, B. Mennucci, H. P. Hratchian, J. V. Ortiz, A. F. Izmaylov, J. L. Sonnenberg, D. Williams-Young, F. Ding, F. Lipparini, F. Egidi, J. Goings, B. Peng, A. Petrone, T. Henderson, D. Ranasinghe, V. G. Zakrzewski, J. Gao, N. Rega, G. Zheng, W. Liang, M. Hada, M. Ehara, K. Toyota, R. Fukuda, J. Hasegawa, M. Ishida, T. Nakajima, Y. Honda, O. Kitao, H. Nakai, T. Vreven, K. Throssell, J. A. Montgomery, Jr., J. E. Peralta, F. Ogliaro, M. J. Bearpark, J. J. Heyd, E. N. Brothers, K. N. Kudin, V. N. Staroverov, T. A. Keith, R. Kobayashi, J. Normand, K. Raghavachari, A. P. Rendell, J. C. Burant, S. S. Iyengar, J. Tomasi, M. Cossi, J. M. Millam, M. Klene, C. Adamo, R. Cammi, J. W. Ochterski, R. L. Martin, K. Morokuma, O. Farkas, J. B. Foresman, and D. J. Fox, *Gaussian 16*, Gaussian Inc., Wallingford CT, **2016**.
[10] Y. Zhao, D. G. Truhlar, *Theor Chem Account* **2008**, *120*, 215–241.
[11] a) G. A. Petersson, A. Bennett, T. G. Tensfeldt, M. A. Al‐Laham, W. A. Shirley, J. Mantzaris, *J. Chem. Phys.* **1988**, *89*, 2193–2218; b) G. A. Petersson, M. A. Al‐Laham, *The Journal of chemical physics* **1991**, *94*, 6081–6090.
[12] F. Weigend, R. Ahlrichs, *Phy. Chem. Chem. Phys.* **2005**, *7*, 3297–3305.
[13] a) P. Hohenberg, W. Kohn, *Phys. Rev.* **1964**, *136*, B864-B871; b) W. Kohn, L. J. Sham, *Phys. Rev.* **1965**, *140*, A1133-A1138.
[14] R. Dennington, T. A. Keith, J. M. Millam, *GaussView, Version 6.0*, Semichem Inc., Shawnee Misssion, KS, **2016**.
